# Systematics and ecology of the Australasian genus *Empodisma* (Restionaceae) and description of a new species from peatlands in northern New Zealand

**DOI:** 10.3897/phytokeys.13.3259

**Published:** 2012-07-03

**Authors:** Steven J. Wagstaff, Beverley R. Clarkson

**Affiliations:** 1 Allan Herbarium, PO Box 40 Landcare Research, Lincoln7640, New Zealand; 2 Landcare Research, Private Bag 3127, Hamilton 3240, New Zealand

**Keywords:** Restionaceae, *Empodisma*, taxonomy, new species, New Zealand

## Abstract

The genus *Empodisma* comprises two species that are ecologically important in wetland habitats. *Empodisma gracillimum* is restricted to south-western Australia, whereas *Empodisma minus* is found in Tasmania, eastern Australia and New Zealand. We sequenced three cpDNA genes for 15 individuals of *Empodisma* sampled from throughout the range of the species. The results support an Australian origin for *Empodisma* sometime during the late Oligocene to early Miocene with more recent dispersal, colonization and diversification in New Zealand. We recovered six genetically distinct maternal lineages: three *Empodisma gracillimum* haplotypes corresponding to the three accessions in our analysis, a wide-ranging *Empodisma minus* haplotype found in eastern Australia and Tasmania, an *Empodisma minus* haplotype found in New Zealand from Stewart Island to approximately 38° S latitude on the North Island, and a distinct haplotype restricted to the North Island of New Zealand north of 38° S latitude. The Eastern Australian and New Zealand haplotypes of *Empodisma minus* were supported by only one cpDNA gene, and we felt the relatively minor morphological differences and the small amount of genetic divergence did not warrant taxonomic recognition. However, we recommend that the northern New Zealand haplotype should be recognized as the new species *Empodisma robustum* and provide descriptions and a key to the species of *Empodisma*. Monophyly of *Empodisma robustum* is supported by all three cpDNA genes. *Empodisma robustum* can be distinguished from *Empodisma gracillimum* and *Empodisma minus* by its robust growth stature and distinct ecology. It is typically eliminated by fire and re-establishes by seed (seeder strategy), whereas *Empodisma minus* and *Empodisma gracillimum* regrow after fire (sprouter strategy).

## Introduction

**Figure 1.**
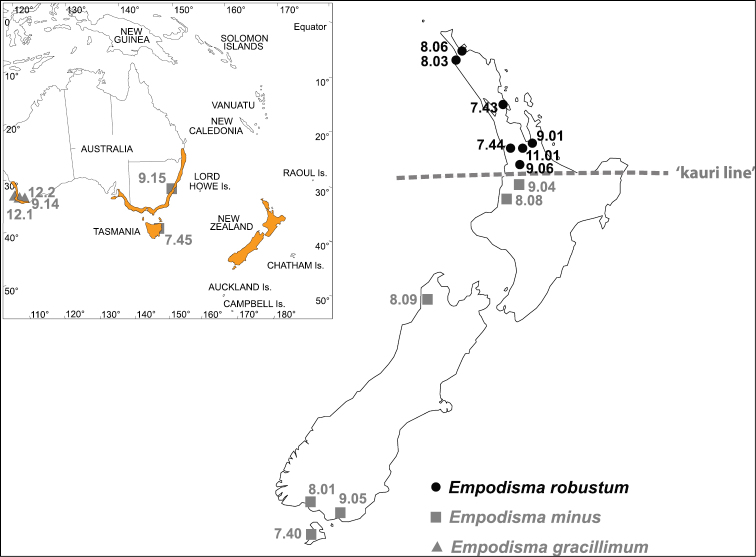
Map showing the generalized distribution of *Empodisma* in Australia and New Zealand and the collection localities of the DNA samples included in our study. The approximate position of the kauri line in New Zealand is shown with a dashed line.

As presently circumscribed, the genus *Empodisma* L.A.S.Johnson & D.F.Cutler (Restionaceae) comprises two species with a widely disjunct distribution in western Australia and south eastern Australia, Tasmania, and New Zealand. *Empodisma gracillimum* (F.Muell.) L.A.S.Johnson & D.F.Cutler is found on the coastal plain from Perth southwards, and along the south coast from Augusta to Albany (Fig. 1), while *Empodisma minus* (Hook.f.) L.A.S.Johnson & D.F.Cutler is found in lowland to alpine zones from Queensland to South Australia, Tasmania and throughout most of New Zealand in New Zealand. They probably diversified in seasonally wet habitats, but exhibit adaptations to seasonal drought, fire and nutrient poor soils (Linder and Rudall 2005).

The species of *Empodisma* are plants of peatlands, particularly raised bogs, blanket bogs, fens, and wet heathlands (Meney and Pate 1999, Johnson and Brooke 1989, Johnson and Gerbeaux 2004). The scientific name is derived from the Greek word for obstacle or hindrance (Johnson and Cutler 1973), and because of their tendency to form dense masses of tangled culms they are also given the common name wire rush. They are rhizomatous perennials with evergreen culms. The horizontal roots branch profusely to form cluster roots (Lamont 1982), i.e. finely divided rootlets with persistent root hairs. The underlying peat is formed mainly from the remains of this densely branched root matrix, which binds litter and bryophytes into the peat (Campbell 1964). The cluster roots retain water like a sponge, up to 15 times their dry weight, and like *Sphagnum* they create acidic conditions (Campbell 1964, 1975, Agnew et al. 1993). In this type of environment incoming rainfall and atmospheric particulates are the major sources of nutrients, which are efficiently removed by the cluster roots of *Empodisma* at the bog surface (Clarkson et al. 2009).

Fire plays an important role in the development of restiad peat bogs in both Australia and New Zealand. For the most part, the species of *Empodisma* are “sprouters” (Pate et al. 1991, Meney et al. 1997, Meney and Pate 1999). In sprouters most of the carbon resources and nutrient elements are allocated towards maintenance and vegetative growth. The underground portions of individual plants are protected and survive fire, and regeneration occurs by the sprouting of new leafy shoots produced from the rhizome system. This contrasts with an obligate “seeder” strategy whereby the plants are killed by fire and re-establish from seed. Seeders generally produce more delicate, less extensive underground rhizome systems and have perennating buds higher in the soil, and without a requirement for nourishing the developing rhizomes, more resources can be allocated to seeds. However, this distinction is not as clear in habitats that experience waterlogged soils during the wet season but have a long intervening dry season, as occurs in much of Australia (Pate et al. 1999).

The taxonomic history of *Empodisma* is complex. The species of *Empodisma* were originally placed in *Calorophus* Labill or *Hypolaena* R.Br. by early taxonomists classifying Restionaceae (Labillardière 1806, Brown 1810, Hooker 1852–1853, Hooker 1857-58, Mueller 1872–1874, Bentham 1878, Cheeseman 1906, Cockayne 1958, Moore and Edgar 1970). The genus *Calorophus* Labill. was originally described by Labillardière (1806). When first described the Tasmanian species *Calorophus elongatus* was the sole member of the genus and is the type. In his first treatment of the flora of New Zealand, Hooker (1853) described a new species of *Calorophus*, *Calorophus minor* Hook.f., based upon Bidwell, Colenso and Lyall specimens. A specimen of *Calorophus minor* collected on the South Island of New Zealand near Nelson by Bidwell was designated as the lectotype by Moore and Edgar (1970). However, in his treatment of the Flora of Tasmania, Hooker (1859) relegated *Calorophus minor* Hook.f. as the variety *Calorophus elongatus* var. *minor* (Hook.f.) Hook.f. Mueller (1872–74) distinguished the plants from western Australia as the distinct species *Calorophus gracillimus* F. Muell., but in Flora Australiensis Bentham (1878) followed Brown’s (1810) earlier treatment, which reduced *Calorophus* to sectional rank within the genus *Hypolaena*. Bentham’s treatment was subsequently followed by Cheeseman (1906) and Cockayne (1958) who recognized the New Zealand plants as *Hypolaena lateriflora* var. *minor* (Hook.f.) Cheesem. However, Moore and Edgar (1970) followed Hooker’s (1859) treatment, adopting the name *Calorophus minor* Hook.f. in their treatment of the Flora of New Zealand, Vol. II. Based on anatomical, morphological and cytological differences among the species of *Calorophus*, Johnson and Cutler (1973) subsequently erected the genus *Empodisma* L.A.S.Johnson & D.F.Cutler to accommodate *Empodisma gracillimum* and *Empodisma minus*.

The specimen upon which Hooker based the name *Calorophus minor* is a small slender plant characteristic of alpine regions found in South Island and Stewart Island of New Zealand. Moore and Edgar (1970) noted that plants at lower elevation in the lowland bogs near Cambridge in the Waikato, northern North Island, were larger and more robust, but based upon study of herbarium specimens they felt the variation was continuous from low to high elevation. We provide evidence for an alternative taxonomic interpretation. The lowland populations of *Empodisma* north of the “kauri line” (the southern limit of *Agathis australis* (D.Don) Lindl. ex Loudon is approximately 38° S latitude in New Zealand) comprise a distinct evolutionary lineage that we here recognize as *Empodisma robustum* S.J.Wagstaff & B.R.Clarkson, sp. nov.

## Methods

### Study group

We conducted a global analysis of 48 members of the Restionaceae to test monophyly of *Empodisma* and its relationships to *Calorophus* and *Hypoleana*. Three genera of Anarthriaceae (*Anarthria* R. Br., *Hopkinsia* W. Fitzg. and *Lyginia* R. Br.) were selected as outgroups. Intraspecific variation within *Empodisma* was assessed by comparing DNA sequences from 18 accessions collected from throughout the range of these species (Fig. 1).

### Data resources

Voucher specimens with their collection locality and GenBank accession numbers are listed in Appendix 1. The aligned data matrices have been submitted in Nexus format to TreeBase matrix accession number http://purl.org/phylo/treebase/phylows/study/TB2:S12748 and the Dryad repository: http://dx.doi.org/10.5061/dryad.94710.

### Morphological analyses

A single set of morphological measurements were taken from each of 76 dried herbarium specimens on loan from AK, CHR, WAIK, WELT and PERTH (abbreviations follow Index Herbariorum). The morphological measurements describe the growth habit and floral structures of *Empodisma* and were confirmed with additional observations from field collections. Because *Empodisma* is dioecious, floral attributes of female flowers were coded as missing on male plants. Also very few specimens had mature fruits, and this attribute was also coded as missing from many specimens. We used GenStat version 8.1.0.152 (supplied by VSN International Ltd., www.vsn-int.com) to illustrate patterns of variation among the characters (listed in Fig. 6) using BOXPLOTS and PRINCIPAL COORDINATES ANALYSIS (PCoA). Principal coordinates analysis depicts relationships among the 91 Operational Taxonomic Units (OTU’s) that comprised our sample. We initially generated a similarity matrix of Euclidean distances then created a two-dimensional ordination. The first axis accounted most of the variation with less variation described by the second axis.

To test the influence of environmental conditions on growth form, we set up a common garden experiment by transplanting individuals (*n=*3) from New Zealand sites representing populations of both large (*Empodisma robustum*)and small (*Empodisma minus*)growth forms to Hamilton, North Island (37°47'S latitude). The sites selected were Torehape (37°18'S), Kopuatai (37°24'S), and Moanatuatua (37°55'S) for the large growth form, and Tongariro (39°16'S), Rangipo (39°22'S), and Awarua (46°33'S) for the small form. Ecological information was summarized from the published literature and unpublished data of BRC.

### DNA extraction, amplification and sequencing

We extracted total DNA from either freshly collected plants or plants dried using silica gel, using a Qiagen DNeasy extraction kit (QIAGEN Pty Inc., Clifton Hill, Victoria, Australia) following the manufacturer’s directions. Three chloroplast-encoded DNA regions were sequenced: *rbc*L, *mat*K and *trn*L. These regions were selected as they have been used previously to resolve relationships within the Restionaceae (Briggs et al. 2000, 2010, Linder et al. 2003, Hardy and Linder 2005, Moline and Linder 2005, Givnish et al. 2010). The genes r*bc*L and *mat*K encode functional proteins, whereas *trn*L encodes part of the gene for Phe–tRNA along with the intervening intron. With very few exceptions chloroplast genes are maternally inherited in flowering plants, so sequence differences correspond to unique haplotypes.

Our PCR amplification and sequencing procedures generally followed those described by Linder et al. (2003) and Hardy and Linder (2005). Excess primers and unincorporated nucleotides were removed from PCR products by a Shrimp Alkaline Phosphatase (GE Healthcare, Global Headquarters, Cahlfont St Giles, UK)/Exonuclease I (Fermentase International Inc, Burlington, ON, Canada) treatment. Sequencing reactions were run on an ABI3730 sequencer (Applied Biosystems, Foster City, CA, USA) by the Allan Wilson Centre Genome Service at Massey University, Palmerston North, New Zealand. In all instances we sequenced both the forward and reverse DNA strands. The sequence contigs were edited using Sequencher 4.8 (Gene Codes Corporation, Ann Arbor, MI, USA).

### Sequence alignment

We used ClustalX (Thompson et al. 1997) to facilitate alignment of the sequences. The sequence alignments for *mat*K and *rbc*L were easily achieved as there no gaps in the *rbc*L and only two gaps in the *mat*K matrix. The gaps in *mat*K occurred in multiples of three and were positioned so as not to disrupt the codon reading frame. The *trn*L sequence alignment across the Restionaceae was more complex, so we used a modification of the sequence profile alignment procedure described by Morrison (2006).

Closely related sequences were initially aligned using the multiple alignment settings, a gap opening penalty of 5, a gap extension penalty of 5, and a delay-divergent-sequences setting of 97%. These ClustalX penalties favour opening gaps rather than substitutions, and they delayed adding the most distantly related taxa in our study. We identified low-scoring segments and exceptional residues, using the quality settings in ClustalX, and reconciled alternative alignments of these short DNA stretches. The final alignments were then visually inspected, and minor adjustments were made manually before conducting the phylogenetic analyses.

Some of the outgroup sequences were not available from GenBank (e.g. a *mat*K sequence was missing for *Chordifex hookeri* (D.I.Morris) B.G.Briggs, but both an *rbc*L and *trn*L sequence were available from GenBank for this taxon). Rather than excluding these taxa, the incomplete data partitions were coded as missing. Many recent empirical and simulated studies suggest that it is possible to include taxa with large amounts of missing data without compromising phylogenetic accuracy. Indeed, increasing both the number of taxa and characters can improve the accuracy of phylogenetic inferences (Weins and Morrill 2011).

### Parsimony and median network analyses

We conducted both parsimony and network analyses of the sequence data sets, using PAUP* 4.0b10 (Swofford 2002) and SplitsTree version 4.8 (Huson 1998, Huson and Bryant 2006). For the parsimony searches we used the settings TBR branch swapping, MULPARS in effect, and RANDOM ADDITION with 1000 replicates. The parsimony characters were unordered and equally weighted. Duplicate trees were eliminated using the “condense trees” option collapsing branches with a maximum length of zero. Congruence of the data partitions was assessed using the Incongruence Length Difference (ILD) test (Farris et al. 1994, 1995) with 100 data partition replicates excluding uninformative sites as suggested by Hipp et al. (2004) and Ramirez (2006). Taxa that were missing one or more of the data partitions were excluded from the ILD test. In the absence of significant conflict, we combined the sequence data sets. Support for clades was estimated by bootstrap (Felsenstein 1985, 1988) with 1000 replications excluding uninformative sites; starting trees were obtained by RANDOM ADDITION with one replication for each bootstrap replicate, TBR branch swapping, and MULPARS in effect. Median networks were constructed using the options add all trivial characters and a minimum support value of 1.

### Bayesian analysis and divergence estimates

Each gene partition was tested for the best substitution model using jModelTest (Posada 2008) with default settings based on the Bayesian Information Criterion (BIC) (Posada and Buckley 2004), averaging over all included parameters in order avoid a bias towards parameter-rich models. The jModelTest comparisons selected TrN + I +G as the best fit model for *rbc*L, TPM1 for *mat*K and TIM1ef for *trn*L. Because the genes *rbc*L and *mat*K encode functional enzymes, we unlinked the substitution rate parameters and the base frequencies across codon positions (1+2), 3. Most of the synonymous mutations occur in the third codon position. The aligned matrices were then prepared as output files for analysis in BEAUti 1.6.1 (part of the BEAST package) and analyzed in BEAST 1.6.1 (Drummond and Rambaut 2007). A Yule prior (Yule 1924) was set for the tree model, together with unlinked relaxed lognormal clock models on the substitution rates for each locus. The MCMC chains were set to run for 90 million generations, logging parameters every 1000 generations. Chain mixing and convergences were checked in Tracer v1.5 (Rambaut and Drummond 2007) with all parameters showing ESS values of > 200. A maximum clade credibility trees was calculated using TreeAnnotator 1.6.1 (Drummond and Rambaut 2007) and a summary with 95% highest posterior density intervals of divergence time estimates was prepared using FigTree v1.3.1 (Rambaut 2009).

### Incorporating uncertainty associated with the fossil record

The earliest verifiable fossils of Poales are from the early Cretaceous (Maastrichtian) deposits dated approximately 115 million years ago (Herendeen and Crane 1995). The Restionaceae are nested within the Poales, so it is unlikely that the age of the restiad lineage is older than 115 million years. The earliest restiad microfossils appear in late Cretaceous deposits in South Africa dated between 64 and 71 million years ago (Scholz 1985) with fossils appearing in progressively younger deposits in Antarctica, Australia, New Zealand and South America (Truswell and MacPhail 2009, Barreda and Palazzesi 2007, McPhail 1997, Scholz 1985, Mildenhall 1980). We attempted to incorporate uncertainty associated with these fossil calibrations by applying a lognormal prior with an offset of 115, a log mean = 2 and a log (Sdev) = 0.5 applied to the root and an offset of 64, a log (mean) = 2 and a log (Sdev) = 0.5 to the node separating the Anarthriaceae from the Restionaceae. These settings provide broad probability distributions between 117.8–134.7 and 66.8–83.7.5, with median values of 118.2 and 71.4 respectively for these calibration points.

## Results

**Figure 2.**
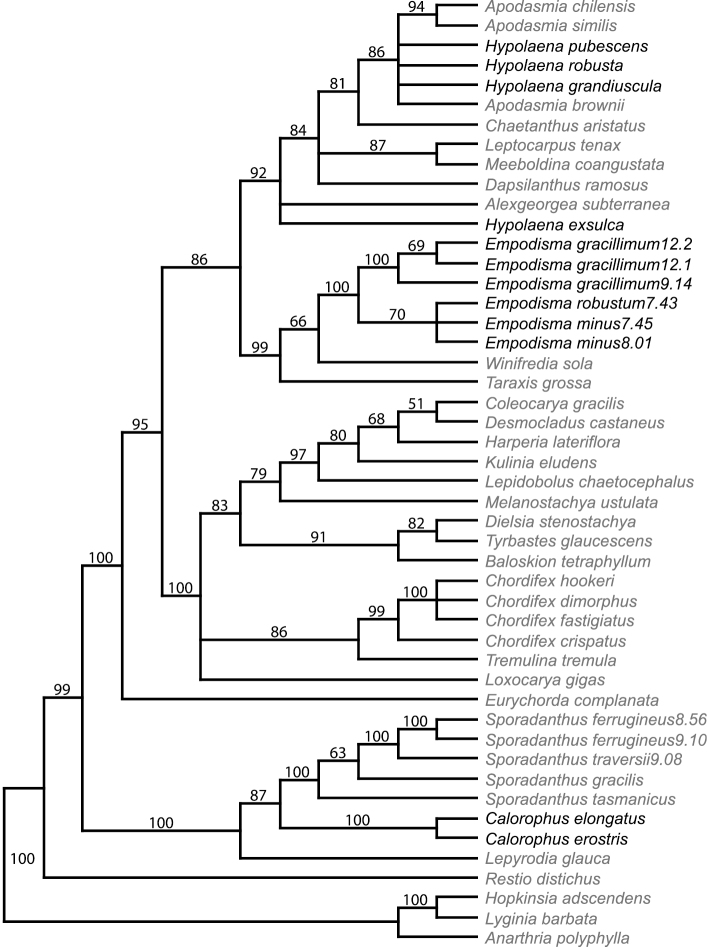
Strict consensus tree. The three species of *Empodisma* (highlighted in bold) emerge as a well-supported clade distinct from *Calorophus* and *Hypolaena*. They were placed in these latter two genera by Moore and Edgar (1970) and Cheeseman (1906). Bootstrap values are provided above the branches

The combined sequence data set comprised three data partitions with a total of 4267 characters; approximately 23% of the total data matrix was comprised of gap or missing data. An ILD test of the three data partitions failed to find significant conflict (*p  *= 1–82/100 = 0.18). A heuristic search with parsimony as the optimality criterion recovered a single island of 180 trees of 2226 steps (Consistency Index (CI) = 0.607 (excluding uninformative characters); Retention Index = 0.788); a strict consensus is shown in Fig. 2. The three gene regions differed in length, the number of variable characters, and the degree to which they resolved and supported phylogenetic relationships.

The strict consensus tree (Fig. 2) agrees with the subfamilial classification of Briggs and Linder (2009). *Restio distichus* is the only representative of Restionoideae (African); *Calorophus*, *Sporadanthus* and *Lepyrodia* (Australian) make up Sporadanthoideae, while the remainder are representative of Leptocarpoideae. In our analysis the three species of *Empodisma* form a well-supported clade (100% bootstrap) with *Empodisma gracillimum* emerging as sister to *Empodisma minus* and *Empodisma robustum*. *Winifredia sola* is weakly supported as sister to *Empodisma*, and *Taraxis grossa* emerges as sister to the *Empodisma*/*Winifredia* clade (99% bootstrap). The two species of *Calorophus* also form a well-supported clade (100% bootstrap), but are distantly related to *Empodisma*, instead emerging as sister (100% bootstrap) to *Sporadanthus*. Likewise *Hypolaena* is also distinct from *Empodisma*. The species of *Hypolaena* are nested within a large clade (92% bootstrap) that includes *Apodasmia* and *Alexgeorgea subterranean* Carlquist.

**Figure 3.**
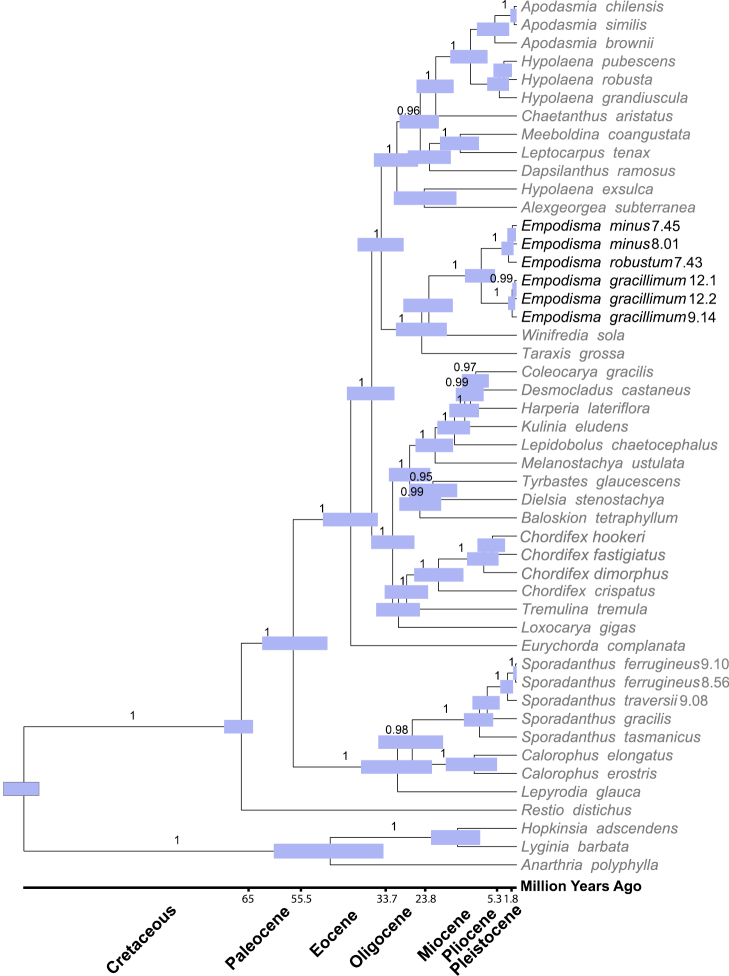
Bayesian chronogram with estimated divergence times. Node error bars are provided in blue showing the 95% highest probability density for the divergence estimates. Posterior probability support values > 97% are given above the branches. A geological time scale is shown at the base of the tree.

The maximum parsimony and Bayesian analyses converged on trees with essentially the same topology, which suggested that the sequence data are robust to the different assumptions associated with these two approaches. Notably, the Bayesian posterior probability values were generally higher than the bootstrap support values, and the chronogram was better resolved (Fig. 3). The 95% highest posterior density estimates revealed substantial uncertainty associated with the divergence estimates, so our results should be viewed as preliminary. The results suggest *Empodisma* diverged from its most closely related ancestor (MCRA), *Winifredia sola* L.A.S.Johnson & B.G.Briggs approximately 21.8 (15.9–28.2) million years ago (mya). *Empodisma gracillimum* diverged at about 8.8 (5.4–12.9) mya and *Empodisma robustum* split from *Empodisma minus* approximately 2.0 (0.8–3.8) mya. Even though the algorithm for dating divergence times was different, the estimates presented here are similar to those obtained by Linder et al. (2003).

**Figure 4.**
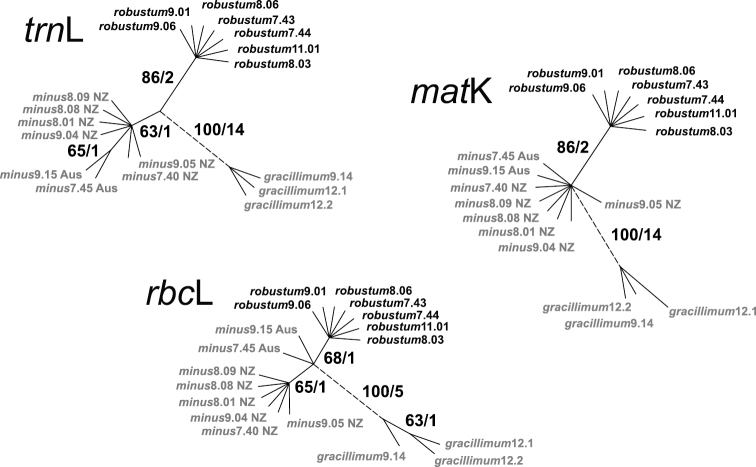
Comparison of median networks from independent analyses of *trn*L, *rbc*L and *mat*K sequences. Bootstrap values / the number of mutations distinguishing each haplotype. are shown beside the branches. The accessions of *Empodisma minus* from New Zealand are indicated NZ and Australia Aus.

A comparison of median networks assessing levels of intraspecific variation in the three species of *Empodisma* is shown in Fig. 4. In each instance only a single parsimony tree was recovered. The *trn*L sequences were the shortest but the most variable. They were 953 nucleotides in length, and of these, 18 substitutions were parsimony informative. Fourteen substitutions supported the *Empodisma gracillimum* lineage with two unique parsimony uninformative substitutions distinguishing *Empodisma gracillimum*9.14 from the other species. One *trn*L character supported the eight accessions of *Empodisma minus* (bootstrap 63%). The split between the Australian (*Empodisma minus*7.45 and *Empodisma minus*9.15) and the New Zealand specimens (*Empodisma minus*8.09, *Empodisma minus*9.05), was supported by one *trn*L character but again with low support (65% bootstrap). Two informative substitutions supported the split between the robust northern New Zealand specimens of *Empodisma* (e.g. *Empodisma robustum*9.06 and *Empodisma robustum*7.44) highlighted in bold and the other specimens of *Empodisma* in our data set (Fig. 4). Further support for this split comes from a 23-base duplication that is absent from *Empodisma robustum* but present in *Empodisma minus* and *Empodisma gracillimum*. This split received 86% bootstrap support in our analysis. The sequences were identical within the seven accessions of *Empodisma robustum*.

The *rbc*L sequences were 1401 nucleotides long; of these, eight characters were parsimony informative and 1393 were constant. The informative characters support four splits in the data (Fig. 4). Five substitutions support *Empodisma gracillimum* with one substitution supporting *Empodisma gracillimum*12.1 and *Empodisma gracillimum*12.2. The third split supports only the diminutive accessions of *Empodisma minus* from New Zealand (65% bootstrap), and one substitution supports the fourth split separating the large lowland form of *Empodisma* (e.g. *Empodisma robustum*7.43 highlighted in bold (68% bootstrap).

By comparison, the *mat*K sequences were 1469 nucleotides long, and of these 16 characters were parsimony informative, 3 variable characters were parsimony uninformative, and 1450 were constant. The informative characters again provided strong support for the split between *Empodisma gracillimum* and the remaining samples in our data (14 substitutions/100% bootstrap) (Fig. 4). *Empodisma gracillimum*12.1 was supported by two unique substitutions and *Empodisma gracillimum*9.14 by one. The split between specimens of *Empodisma robustum* from northern New Zealand and *Empodisma minus* was supported by 2 substitutions / 86% bootstrap. The sequences within these latter two groups were identical.

**Figure 5.**
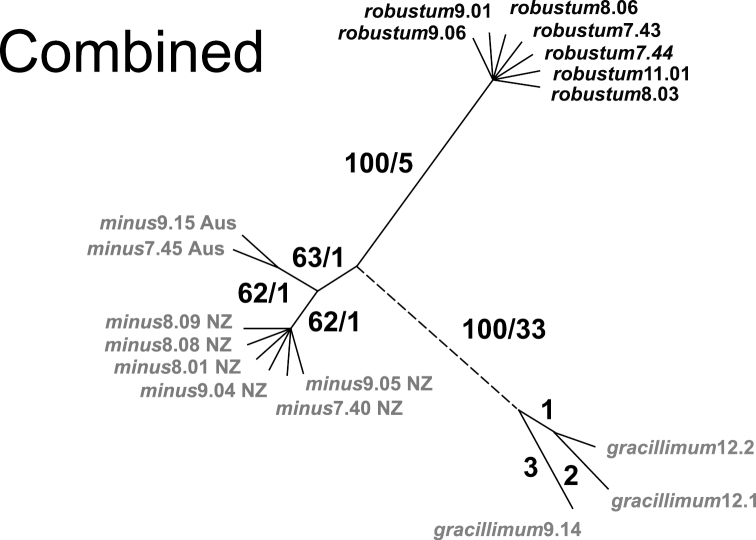
Unrooted parsimony tree from a combined analysis of the three sequence partitions. Six distinct cpDNA haplotypes are supported. Bootstrap values / the number of mutations distinguishing each haplotype are shown beside the branches. The accessions of *Empodisma minus* from New Zealand are indicated NZ and Australia Aus.

The Incongruence Length Difference test failed to reveal significant incongruence (*p* = 1–1/100 = 1.00) among the three independent data sets, so we pooled them. An analysis of the combined data recovered a single maximum parsimony tree; an unrooted phylogram is shown in Fig. 5 (Consistency Index, excluding uninformative characters = 1.00, Retention Index = 1.00). The combined analysis provided strong support for clades corresponding to the western Australian endemic, *Empodisma gracillimum* (33 substitutions / 100% bootstrap) and the robust northern New Zealand plants (e.g. *Empodisma robustum*9.06 and *Empodisma robustum*9.01 (five substitutions / 100% bootstrap), but weak support for *Empodisma minus* (1 substitution / 63% bootstrap).

We distinguished six distinct cpDNA haplotypes within the three species of *Empodisma*. Each accession of *Empodisma gracillimum* was distinguished by one or more substitutions and constituted three unique haplotypes. The two sequences of *Empodisma minus* from eastern Australia and Tasmania were identical and comprised the fourth haplotype (see also Figure 1). These were distinguished from a fifth haplotype comprising the *Empodisma minus* accessions from New Zealand ranging from Stewart Island to approximately 38° S latitude on the North Island. The sequences of *Empodisma robustum* restricted to the North Island of New Zealand north of 38° S latitude comprised a sixth haplotype.

We observed also a substantial degree phenotypic variation within the species of *Empodisma* especially in those characters that describe growth habit, e.g. culm height, internode distance, sheath length and leaf length. However, when grown together in common garden experiments in Hamilton, the two New Zealand species, *Empodisma robustum* and *Empodisma minus*, retained their distinctive growth forms, which suggests there is a genetic component to the pattern of morphological variation.

**Figure 6.**
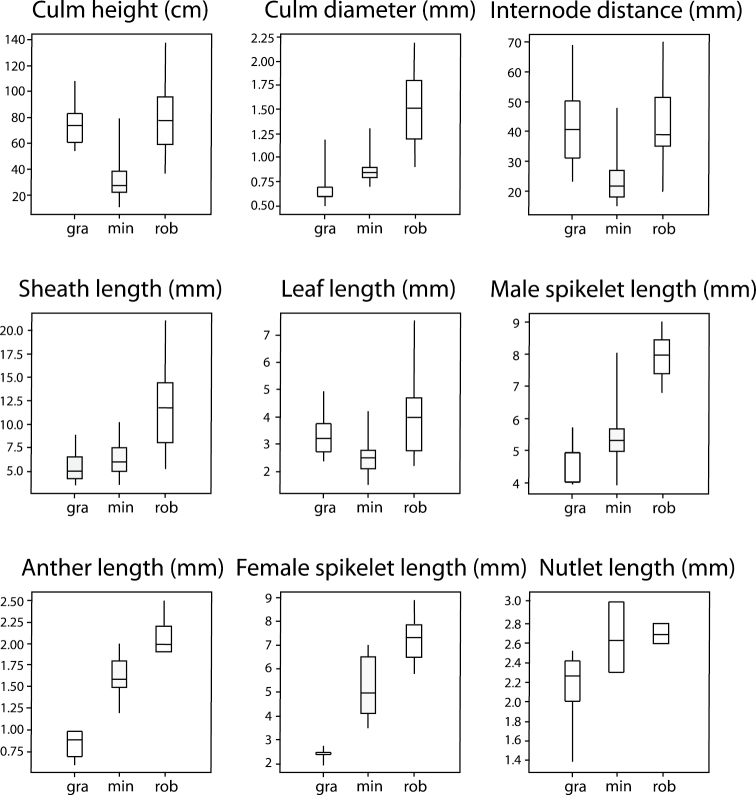
Box plots illustrating patterns of morphological variation among the species of *Empodisma*. The box spans the interquartile range of the values in the variate. The middle 50% of the data lie within the box, with a line showing the median. The whiskers extend beyond the ends of the box as far as the minimum and maximum values.

*Empodisma robustum* is generally a larger more robust plant, which ranges in height from 0.4 to over 1.3 meven taller in supporting vegetation, whereas *Empodisma minus* approaches 0.8 m in lowland bogs in Queensland, but in southern latitudes and alpine environments the plants are dwarfed, barely reaching 0.3 m (Fig. 6). *Empodisma gracillimum* is similar in height to *Empodisma robustum*, but the culms are light green in colour and more delicate; usually they are less than 0.7 mm in diameter. The culms of *Empodisma robustum* are dark green and broader, in some individuals approaching 2.2 mm in diameter. The culms of *Empodisma minus* are also dark green, but they are seldom greater than 1.0 mm in diameter. Internode distances also vary substantially among the three species; the distances are greater in *Empodisma robustum* and *Empodisma gracillimum* ranging from 20.0 to 70.0 mm in *Empodisma robustum* and from 25.0 to 80.0 in *Empodisma gracillimum* in contrast to *Empodisma minus* which ranges from 15.0 to 48.0 mm (Fig. 6). The leaf sheaths of *Empodisma robustum* also tend to be longer, ranging from 5.2 to 21.0 mm, whereas the leaf sheaths of *Empodisma gracillimum* range from 3.5 to 9.3 mm in length andfrom 3.5 to 10.2 mm in *Empodisma minus*. The leaves of *Empodisma robustum* are also longer, ranging from 2.2 to 7.55 mm, while the leaves range in length from 2.4 to 5.0 mm in *Empodisma gracillimum* and from 1.5 to 4.2 mm in *Empodisma minus*. The floral structures of *Empodisma robustum* are substantially longer than those of *Empodisma minus*. In contrast, the inflorescences of *Empodisma gracillimum* are smaller and more delicate then either *Empodisma robustum* or *Empodisma minus* (Fig. 6). The male spikelet of *Empodisma robustum* ranges from 6.8 to 9.0 mm in length, whereas *Empodisma minus* ranges from 3.9 to 8.0 mm and *Empodisma gracillimum* from 4.0 to 5.8 mm. The anthers in *Empodisma robustum* range from 1.9 to 2.5 mm in length, 1.2 to 2.0 in *Empodisma minus*, and 0.6 to 1.0 mm in *Empodisma gracillimum*. The female spikelets in *Empodisma robustum* ranges from 5.8 to 8.9 mm in length, 3.5 to 7.0 mm in *Empodisma minus* and 1.5 to 2.4 mm in *Empodisma gracillimum*. While few of the herbarium specimens that we examined had mature fruits, fruits from *Empodisma robustum* ranged from 2.6 to 2.8 mm in length, 2.3 to 3.0 mm in *Empodisma minus*, and 1.4 to 2.5 mm in *Empodisma gracillimum*.

**Figure 7.**
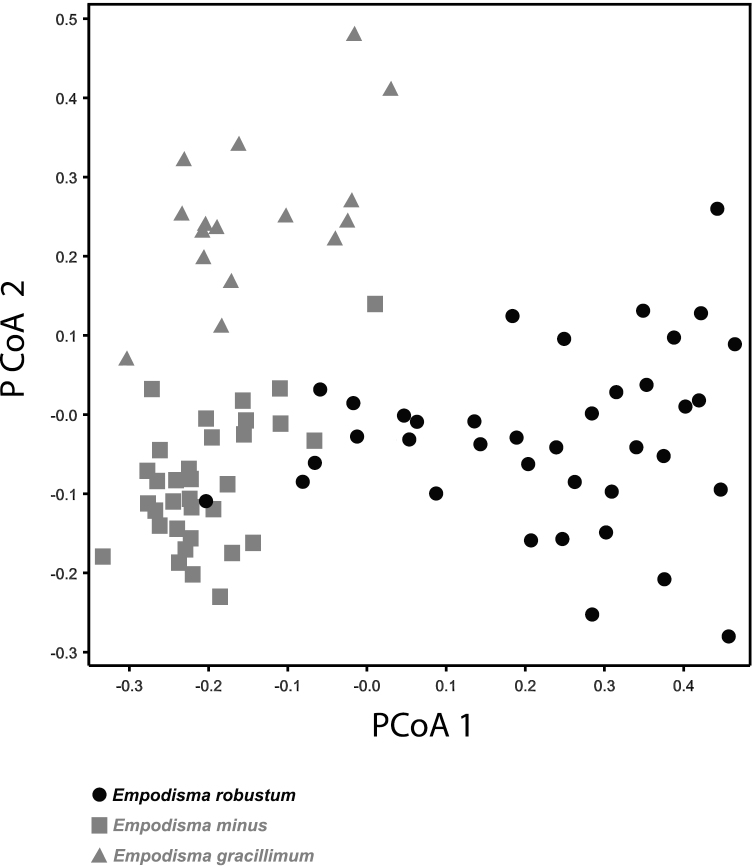
Principal coordinates ordination depicting patterns of overall similarity among the 74 OTEs that comprised our morphological sample. The first PC axis accounted for 58.3% of the variation in our sample and the second PC axis accounted for 23. 4% of the variation.

The first PCoA axis accounted for 52.4 % of the variation in our sample and the second 23.4% (Fig. 7). The PCoA ordination separated *Empodisma gracillimum* primarily on the second axis but there was some overlap among the outliers of *Empodisma robustum* and *Empodisma minus* on the first axis. The greatest spread among the OTU’s was observed in *Empodisma robustum* and *Empodisma gracillimum*; this might reflect their taller more scrambling growth habit. With the exception of one outlier from Queensland, specimens of *Empodisma minus* are more tightly grouped. Several of the specimens of *Empodisma minus* were collected in the high mountains or in lowland bogs at more southerly latitudes. The stature of these plants may be more constrained by the harsh environments that they inhabit.

## Discussion

DeQueiroz (2007) proposed a unified species concept based on the single common element of most contemporary species definitions. He suggested that species are separately evolving metapopulation lineages. A metapopulation is a series of connected populations. A lineage implies an ancestor–descendant relationship. Species have a number of emergent biological properties, but these often arise at different times during the speciation process, and taxonomists may place a different emphasis on the biological properties that are used to define species. Incipient species may occupy a new adaptive zone, but this frequently precedes reproductive isolation and fixed morphological differences. The lowland plants of *Empodisma* from northern New Zealand exhibit many of these emergent properties. We feel the evidence is sufficient to justify recognizing the northern New Zealand plants as a distinct species and propose the name *Empodisma robustum* S.J.Wagstaff & B.R.Clarkson sp. nov., which reflects its robust stature.

There is considerable uncertainty associated with estimating divergence times in Restionaceae. The fossil calibrations rely entirely upon microfossils and their affinities to extant genera are not clear. The divergence estimates presented here will undoubtedly be refined as more complete fossils are discovered. Nonetheless our preliminary findings suggest that *Empodisma* evolved in Australia during the mid Oligocene / early Miocene between 28–16 mya (Fig. 3). This was a time of warm, equitable environmental conditions. Crisp and Cook (2007) suggest members of the Restionaceae may have at one time been more widely distributed in Australia, but a rapid succession of marine incursions, the onset of aridity and the origin of the Nullarbor Plain during the Miocene beginning about 13–14 mya created climatic and edaphic barriers that isolated lineages in the southwestern and southeastern sclerophyll biomes in Australia. The split between *Empodisma gracillimum* and *Empodisma minus* (13–5 mya) roughly coincided with these environmental changes. Colonization and diversification in New Zealand occurred more recently and was perhaps induced by uplift of the Southern Alps during the Pliocene and episodes of glaciation during the Pleistocene. The split between *Empodisma minus* and *Empodisma robustum* (4–0.8 mya) spans this time frame.

The sequencingresults also differentiate the populations of *Empodisma robustum* from northern New Zealand from the Australian or New Zealand populations of *Empodisma minus* (Fig. 1), and this split was independently supported by each of the chloroplast DNA regions that we surveyed (Fig. 4). *Empodisma robustum* comprises a distinct evolutionary lineage united by six synapomorphies (Fig. 5). In contrast, the plants from mainland Australia and Tasmania are very similar to the diminutive lowland plants of *Empodisma minus* from southern New Zealand, each haplotype is distinguished only by a single mutation and the plants are found in similar habitats. The three genes that we sequenced were encoded in the chloroplast and are probably maternally inherited. Interestingly, there is no homoplasy in the data. Nonetheless, it is conceivable that the chloroplast gene tree is not compatible with the species tree. Gene convergence, introgression hybridization and/or incomplete lineage sorting could result in incompatible phylogenetic signals. A degree of reproductive isolation is necessary for these mutations to become fixed, which suggests *Empodisma robustum* and *Empodisma minus* have been reproductively isolated, perhaps since the Pleistocene. Within the northern haplotype, the sequences are identical. Although the sample is small, the absence of unique mutations (autapomorphies) suggests gene flow is unrestricted among the northern populations of *Empodisma robustum*. Historical rates of gene flow have traditionally been estimated indirectly from the number of fixed alleles in subpopulations relative to the total population (Sork et al. 1999, Slatkin and Maddison 1981).

Contrary to the observations of Moore and Edgar (1970), our results show that in New Zealand the pattern of morphological variation in *Empodisma minus* is not continuous from low to high elevation; rather two morphologically distinct New Zealand species can be readily distinguished. This interpretation is based upon recent collections from throughout the range of the species. *Empodisma robustum* differs by its more robust growth habit. The stature of *Empodisma minus* diminishes with increasing latitude and altitude. Though they have distinct haplotypes, the diminutive plants of *Empodisma minus* from New Zealand are morphologically very similar to plants from Tasmania, though plants from 0.4 to 1 m tall are noted from eastern Australian. We examined a specimen from Queensland that was 0.8 m tall. The culm diameter of 0.8 mm placed it within the range of the New Zealand accessions, but this plant was an outlier in the PCoA ordination (Fig. 7).

Hooker (1859) stated that the New Zealand plants could be distinguished from the Australian plants by their more woolly sheaths with erect apices. After close inspection we felt this was a relatively minor morphological difference, and considering the low bootstrap support and the small amount of genetic divergence, we did not consider these differences worthy of taxonomic recognition. *Empodisma gracillimum* emerges as sister to *Empodisma minus* and *Empodisma robustum* and is separated by 36 unique nucleotide substitutions. With its delicate light green culms, unbranched multicellular hairs on the rhizome, and pedicellate female flowers, *Empodisma gracillimum* is morphologically very distinct from *Empodisma robustum* and *Empodisma minus*.

The three species of *Empodisma* also have distinct ecological and distributional differences.

### Ecology of *Empodisma robustum*

*Empodisma robustum* is restricted to the region north of 38° S latitude on the North Island of New Zealand. This phytogeographical boundary has long been recognized by New Zealand ecologists and marks the southernmost range of many species, most notably *Agathis australis*, but also bog associates such as *Sporadanthus ferrugineus* de Lange, Heenan & B.D.Clarkson, *Dianella haematica* Heenan & de Lange , *Dracophyllum lessonianum* A.Rich.,* Anzybas carsei* (Cheeseman) D.L.Jones & M.A.Clem., and *Lycopodiella serpentina* (Kunze) B.Øllg. This region is the warmest in New Zealand and is rich in endemic species (McGlone 1985). The pre-European vegetation in this part of New Zealand consisted primarily of warm-temperate forests and restiad peatlands.

*Empodisma robustum* is a mid- to late-successional species of restiad raised bogs in the lowland zone of northern North Island (Clarkson et al. 2004b). In the oldest bogs it forms a dense layer of sprawling, intertwined wiry stems 1–1.8 m in height, overtopped by swards of the bamboo-like *Sporadanthus ferrugineus* (also Restionaceae) up to 2.5 m tall (de Lange et al. 1999). Other canopy associates include the heath shrubs *Leptospermum scoparium* J.R.Forst. & G.Forst., *Epacris pauciflora* A.Rich., and *Dracophyllum lessonianum*, the sedges *Baumea teretifolia* (R.Br.) Palla and *Schoenus brevifolius* R.Br., and the fern *Gleichenia dicarpa* R.Br.* Sphagnum cristatum* Hampe is also present, but does not thrive in the shade of the taller restiads. These bogs were initiated in the post-glacial period (after 14 000 years BP; Newnham et al. 1995, McGlone 2009), and typically formed extensive domes covering up to 15 000 ha, with peat 10–12 m deep (Cranwell 1939). However, widespread drainage and development into pasture in the early to mid-1900s has confined the *Sporadanthus*–*Empodisma robustum* association to three sites in the Waikato: Torehape, Kopuatai, and Moanatuatua (de Lange et al. 1999, Clarkson 2002). Elsewhere in the northern North Island, *Empodisma robustum* occurs in fens and young restiad bogs (Johnson and Brooke 1989, Johnson and Gerbeaux 2004, Hodges and Rapson 2011), and gumland heaths (Clarkson et al. 2011). Apart from *Sporadanthus ferrugineus*, the species associated in these younger/shallower peat systems are similar to those listed above.

*Empodisma robustum* is the key species in the fen–bog transition (Clarkson et al. 2004b, Hodges and Rapson 2011) during the development of restiad raised bogs north of 38° S latitude. It is tolerant of a wide environmental range, establishing early in relatively fertile fens (dominated by *Gleichenia dicarpa*,sedges and heath shrubs) to initiate raised-bog development, and persists in significant amounts in low-nutrient, late-successional phases. It is the main peat former, with its dense surface layer of cluster roots that have high water-holding capacity (Campbell 1964), high resistance to decay (Kuder et al. 1998), and similar base-exchange properties to *Sphagnum* (Agnew et al. 1993). The presence of an initial *Empodisma robustum* phase has been shown to be a precursor to the establishment of *Sporadanthus ferrugineus*, which becomes the physiognomic dominant in late-successional restiad raised bogs (Clarkson et al. 2004a).

The development of raised bogs is constrained by a delicate water balance. They typically form in regions with moderate to high rainfall, cool summers, poor drainage, and isolation from flowing water (McGlone 2009). The warm-climate northern North Island lowlands thus appear unsuitable for raised bogs, having frequent dry summers with extended water deficits and a negative annual water balance (McGlone 2009). However, bogs with dense *Empodisma robustum* canopies have much lower evaporation rates than other wetland plant communities (Thompson et al. 1999). This is likely due to the high water-use-efficiency properties of *Empodisma robustum*, namely reducing water loss by physiological controls of stomatal opening, having reduced scale-like leaves, and a dense mulch of decay-resistant culms, which protects the thick water-retaining root matrix at the bog surface (Campbell and Williamson 1997).

Because the raised-bog surface is isolated from the influence of groundwater and surface runoff, plants receive their water and nutrients from rainfall. They typically have very low levels of plant nutrients, particularly of nitrogen and phosphorus (Damman 1978, Clarkson et al. 2005). It has been shown that *Empodisma robustum* is able to co-exist with the less nutrient demanding late-successional species *Sporadanthus ferrugineus*, by occupying different root zones (Clarkson et al. 2009). *Empodisma robustum* forms a thick layer of cluster roots that overlie the deeper roots of *Sporadanthus ferrugineus*, allowing preferential access to dissolved nutrients in rainfall.

Despite their saturated substrates, naturally occurring fires have been well documented in New Zealand peatlands (Newnham et al. 1995, McGlone 2009), and the frequency of fires has increased dramatically in recent times owing to land clearance by Polynesians and more widely by European settlers. Clarkson (1997) studied recovery from fire in two restiad raised bogs characterised by *Empodisma robustum* at Whangamarino and Moanatuatua in Waikato. The populations of *Empodisma robustum* were eliminated by fire and had to re-establish from seed, taking 4 years to achieve dominance at the two sites. Some minor resprouting was observed in localised pockets at Whangamarino (R.M. Irving, pers. comm., 1993) but recovery after fireis mostly via seed. *Empodisma robustum* has an erect rhizome, and its roots spread horizontally just below the surface of the bog, so its root system is susceptible to fire damage. Those species with rhizomes that penetrated deeply into the substrate, e.g. sedges, resprouted rapidly after fire and dominated in the first few years post-fire, before the restiads resumed pre-fire height and cover (*Empodisma robustum* within 6 years and, where present, *Sporadanthus ferrugineus* within 12 years).

### Ecology of *Empodisma minus*

*Empodisma minus* in New Zealand is also a mid- to late-successional wetland species. It dominates fens, blanket bogs, raised bogs, and pakihi heaths in coastal to alpine areas between 38° S latitude in the North Island and 48° S on Stewart Island, being particularly common in Westland and Southland. It is absent from Chatham Island. The vegetation is typically a dense, springy carpet of *Empodisma minus*, averaging 40 cm tall, associated with heath shrubs (*Leptospermum scoparium*, *Dracophyllum oliveri* Du Rietz, *Dracophyllum prostratum* Kirk), sedges (*Baumea teretifolia*, *Baumea tenax* (Hook.f.) S.T.Blake), the ferns *Gleichenia dicarpa* and *Gleichenia microphylla* R.Br., the tussock grass *Chionochloa rubra* Zotov, sundews (*Drosera* spp.), and *Sphagnum cristatum* moss.

Many of the ecological properties of *Empodisma robustum* also apply to *Empodisma minus*. For example, *Empodisma minus* forms peat via its cluster roots, although these are smaller and less dense than in *Empodisma robustum*. It is also a key species in the fen–bog transition, particularly *Chionochloa rubra*-dominated fens (Hodges and Rapson 2011), and is the major peat former, except in very wet areas favoured by *Sphagnum* mosses (Rigg 1962, Burrows and Dobson 1972, Mark and Smith 1975, Whinam and Kirkpatrick 1995).

*Empodisma minus* resprouts after fire (Timmins 1992, Johnson 2001), and has probably become more common at the expense of woody species, because of land-clearance fires (McGlone 2009). Studies in vegetation recovery after fire in the far south of New Zealand at Eweburn Bog (Timmins 1992) and Awarua Bog (Johnson 2001) first noted resprouting (and some seed establishment) a few months after fire, but recovery was extremely slow and *Empodisma minus* cover was still increasing after 4.5 years at Eweburn and 10 years at Awarua. However, after 40 years there was little difference observed in the cover of *Empodisma minus* in the burnt and unburnt areas in a south Westland mire (Merton 1986). The magnitude of vegetation damage (and hence recovery) is determined by the intensity of the fire, which is influenced by site conditions such as water table depth, fuel build-up and climate (Timmins 1992, Clarkson 1997). In cooler, wetter regions, e.g. southern South Island, fires are likely to be less intense than fires in the northern North Island, which may favour the sprouter recovery strategy over the seeder strategy.

In Australia, *Empodisma minus* occurs in all states apart from Western Australia and Northern Territory, being concentrated in south-eastern Australia. It grows in similar habitats to New Zealand, i.e. fens and bogs, and seasonally or permanently inundated heaths, swamps and stream margins (Campbell 1983, Meney and Pate 1999, Whinam and Hope 2005) from sea level to alpine areas. *Empodisma minus* is most abundant at higher elevations, e.g. eastern Victoria highlands, and in cooler, wetter climates, e.g. Tasmania. Common associates include heath shrubs (*Richea continentis* B.L.Burtt,* Baeckea gunniana* Schauer,* Leptospermum lanigerum* Sol. ex Aiton) Sm.,* Epacris* spp.), sedges (*Carex gaudichaudiana* Kunth,* Carpha alpina* R.Br.), ferns (*Gleichenia alpina* R.Br.,* G. dicarpa*), restiads, e.g. *Baloskion australe* (R.Br.) B.G.Briggs & L.A.S.Johnson, the monocotyledonous herb *Astelia alpina* R.Br., and *Sphagnum cristatum* moss. In lowland zones on the Australian mainland, e.g. eastern Australia, the more arid climate is not conducive to the formation of extensive raised peat bogs characteristic of lowland New Zealand (Campbell 1995). Conditions are suitable for *Empodisma* root growth and peat accumulation only during the wet season (usually winter). Dry conditions during the remainder of the year check root production and accelerate decomposition, resulting in only shallow deposits of peat. Associates in these warmer areas include the grass tree *Xanthorrhoea fulva* (A.T.Lee) D.J.Bedford, and heath and heath-like shrubs including *Sprengelia sprengelioides* (R.Br.) Druce,* Persoonia virgata* R.Br. and *Boronia falcifolia* A.Cunn. ex Endl. Recovery of *Empodisma minus* after fire is rapid. In the Victoria highlands, abundant resprouted plants were noted within a few weeks of being burnt (Walsh and McDougall 2004), with new shoots from basal resprouts being several centimetres long within a month (McDougall 2007). *Empodisma minus* cover had returned to prefire levels within two years of burning and had continued to increase considerably by 17 years post-fire (Wahren and Walsh 2000). However, recovery of community composition to pre-fire levels may take many years because competition from *Empodisma* may impede the establishment and growth of more fire-sensitive species such as *Richea continentis* and *Epacris* spp.

### Ecology of *Empodisma gracillimum*

*Empodisma gracillimum* is endemic to Australia. It is restricted to the coastal plain from Perth southwards, and along the south coast from Augusta to Albany (Meney and Pate 1999). This region receives the greatest amount of rainfall in the Southwest Australian Floristic Region (Hopper and Gioia 2004). *Empodisma gracillimum* inhabits seasonally or permanently inundated swamps, woodlands and stream margins on nutrient poor, peat or sandy peat soils (Meney and Pate 1999). It is locally abundant, forming dense masses up 1.5 m high, is often associated with *Beaufortia sparsa* R.Br., *Leptocarpus* sp., and *Baumea rubiginosa* (Sol. ex G.Forst.) Boeckeler, and is often surrounded by tall shrubs such as *Agonis linearifolia* (DC.) Sweet, *Agonis parviceps* Schauer, Homalospermum firmum Schauer, *Hakea linearis* R.Br., *Callistemon glaucus* Sweet, and the woodland species *Eucalyptus marginata* D.Don ex Sm. and *Eucalyptus calophylla* Lindl. Flowering occurs in the spring and summer with a prolonged seed maturation of 10 to 12 months. The vegetation of the Southwest Australia Floristic region is subject to frequent fires, and *Empodisma gracillimum* employs a sprouter recovery strategy following a fire (Meney et al. 1997).

## Conclusions

The three species of *Empodisma* form a well-supported clade. The clade diverged during the early Miocene, which was a period of equitable environmental conditions in Australia. A rapid succession of marine incursions, the onset of aridity in Australia, and origin of the Nullarbor Plain during the mid to late Miocene created barriers that isolated the southwest Australian endemic *Empodisma gracillimum* from the southeastern Australian *Empodisma minus*. Dispersal, colonization and speciation in New Zealand occurred more recently, coinciding with the uplift of the Southern Alps during the Pliocene and episodes of glaciation during the Pleistocene. Genetic, morphological and ecological evidence supports the separation of *Empodisma minus* into two species, *Empodisma minus* and *Empodisma robustum*. The split between *Empodisma minus* and *Empodisma robustum* is unambiguous and independently supported by the three cpDNA regions that we surveyed. *Empodisma robustum* is distinguished by six unique nucleotide substitutions and a 23-base duplication. It is a taller, more robust plant that is typically killed by fire and confined to lowland regions north of 38° S, whereas *Empodisma minus* is smaller, resprouts after fire, and occurs in alpine and lowland areas south of 38° S. The western Australian species *Empodisma gracillimum* emerges as sister to *Empodisma minus* and *Empodisma robustum*. It is geographically isolated and can be readily distinguished by its fine light green culms, shorter leaf sheaths and pedicellate female flowers. This last character appears to be a distinctive feature of the species.

## Taxonomy

### Empodisma

L.A.S.Johnson & D.F.Cutler

http://species-id.net/wiki/Empodisma

#### Type species.

***Empodisma minus*** (Hook.f.) L.A.S.Johnson & D.F.Cutler

#### Description.

Perennial herbs forming dense tangled masses, dioecious. Rhizomes stout up to 8.0 mm diam., covered with light brown, imbricate, scale-like sheaths and very thick tufts of brown hairs. Roots crowded, densely covered with persistent root hairs. Culms evergreen, hollow, dark to light green, profusely branching. Lamina reduced, awl-shaped, persistent, light green when young maturing dark brown, strongly reflexed from the leaf sheath. Leaf sheaths open, but overlapping and closely appressed, borne at short intervals, straw-coloured early in the season maturing dark brown, mouth ciliate with a tuft of woolly white hairs. Spikelets unisexual, borne in ultimate branch systems produced in second or third year, sessile or on short pedicels. Glumes imbricate; bracteoles lacking. Perianth segments 6, narrowly ovate almost hyaline. Male spikelets with 1–6 flowers, sessile to shortly pedicellate. Stamens 3, exserted beyond the perianth segments; filaments uniform; anthers linear oblong, dorsifixed, 1-celled, dehiscence along longitudinal slits, straw-coloured. Female spikelets solitary, each spikelet with 1– rarely 2 – flowers sessile to pedicillate. Ovary 1-celled; style branches 2 or 3, filiform, deciduous. Ovule solitary, pendulous. Fruit 1-seeded nut, ovoid with a thick and swollen base. 2n = 24. Fruit development is protracted with the seeds maturing in the following winter or early spring.

#### Key to species of *Empodisma*

**Table:** 

1	Robust plants forming dense tangled thickets; culms > 1 mm in diameter at base of the plant; sheaths mostly >7.5 mm long; spikelets > 6.0 mm long	*Empodisma robustum*
–	Slender and/or diminutive plants; culms mostly < 1 mm in diameter, sheaths mostly < 7.5 mm long; spikelets generally < 6.0 mm	2.
2	Culms dark green, 18–80 cm in height (some plants rarely to 1.2 m in Eastern Australia); female spikelets sessile to shortly pedicellate	2. *Empodisma minus*
–	Culms light green, 55–130 cm in height; spikelets borne on pedicels up to 20 mm long	3. *Empodisma gracillimum*

### 1. Empodisma robustum

Wagstaff & B.R.Clarkson sp. nov.

urn:lsid:ipni.org:names:77120446-1

http://species-id.net/wiki/Empodisma_robustum

#### Holotype.

**Figure 8.**
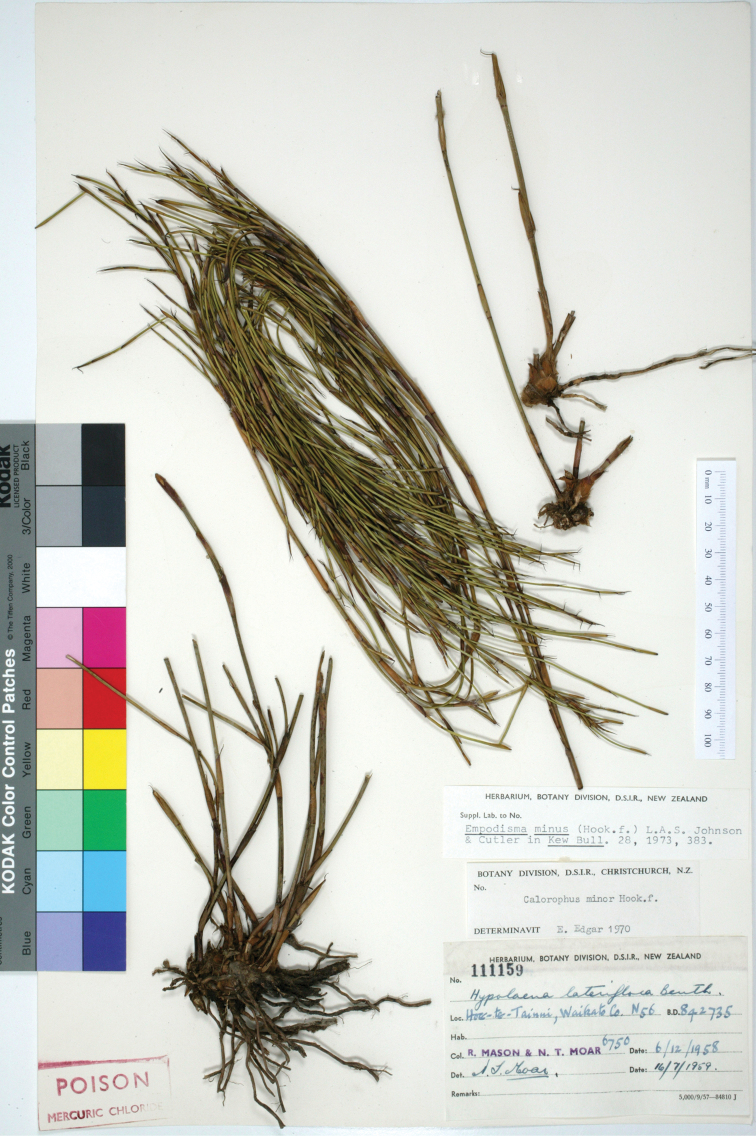
Type of *Empodisma robustum*, N56 R. Mason, N.T. Moar 6750, 6/12/1958, CHR11159.

(Fig. 8) New Zealand, Waikato, Hoe-O-Tainui, R. Mason, N.T. Moar 6750, 6/12/1958, CHR11159.

#### Etymology.

robustum describes the robust stature of *Empodisma robustum*.

#### Description.

**Figure 9.**
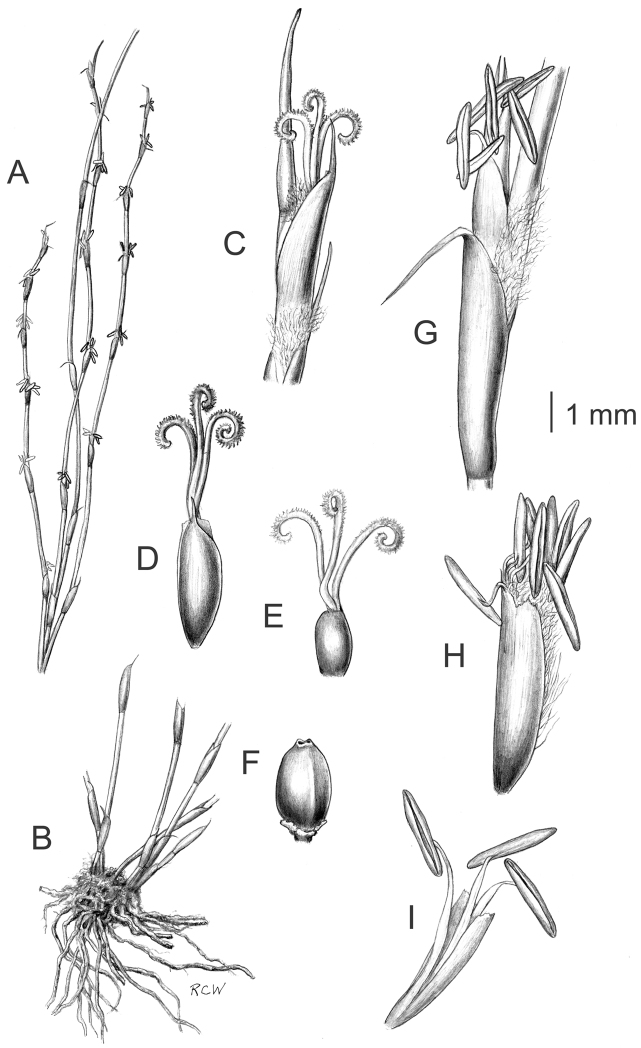
Morphological characteristics of *Empodisma robustum*. **A** Vegetative shoot with attached leaves and flowers (actual size) **B** Rhizomes with emerging vegetative shoots (2.5× actual size) **C** Vegetative shoot with attached pistillate flower **D** Pistillate flower with attached bracts **E** Gynoecium **F** Mature nut **G** Vegetative shoot with attached staminate spikelet **H** Staminate flower with attached bracts **I** Staminate flower. Scale bar = 1 mm.

Culms dark green, 38–139 cm in height (reportedly > 200 cm when supported by associated shrubs), 0.9–2.2 mm in diameter at the base, branching profusely. Leaf sheaths open, closely appressed, 5.2–21.0 mm in length, borne at intervals of 20.0–70.0 mm, light green to light brown early in the season maturing dark brown; mouth ciliate with a tuft of woolly white hairs. Lamina strongly reflexed from leaf sheath, 2.2–7.5 mm long, light green when young maturing dark brown. Spikelets brown; male spikelet 6.8–9.0 mm long, anthers 1.9–2.5 mm; female spikelet 5.8–8.9 mm; nutlets dark brown approximately 2.7 mm long. Flowering Aug.–Oct. See Figure 9.

#### Comments.

Many herbarium specimens of *Empodisma robustum* include only the upper portion of the plant. These specimens may be difficult to distinguish from the larger specimens of *Empodisma minus*. Quality specimens should include a rhizome and the base of the culms, from which the distinguishing measurements are taken. Most collections of *Empodisma robustum* are either sterile or male, and the few females generally lack mature fruits. A chromosome count of 2n=24 was reported from plants collected at Moanatuatua Bog (Briggs 1966, Johnson and Cutler 1973).

#### Representative specimens.

New Zealand, Lake Tangonge, H. Carse, H.B. Mathews, 25 Oct 1920, CHR295186; New Zealand, Moanatuatua Bog, W.F. Harris, 20 Nov 1951, CHR85625; New Zealand, Motutangi Swamp, T. Seymour, 15 July 1976, CHR287072; New Zealand, Tauhei, H. Carse, Aug 1925, CHR295191; New Zealand, Opuatia Bog, immature ♀ flowers J.T. Taylor, 27 July 1987, WAI8520; New Zealand, Torehape, not flowering, R.H. Chitty, WAI3280; New Zealand, Moanatuatua Bog, not flowering, R. Thompson, 3/77, WAI2099; New Zealand, Moanatuatua Bog, immature flower buds, H. Beaton 3/77, WAI2098; New Zealand, Kopouatai Peat Dome, immature flower buds, P.J. de Lange, 14 Mar 1988, WAI9008; New Zealand, ♂ in flower, R. Irving, M. Skinner, 12 Oct.1983, WAI 422; New Zealand, Tairua Ecological District, not flowering, B.R. Clarkson, 3 Feb 1998, WAI 16755; New Zealand, MoanatuatuaBog, not flowering, H.J. Beaton, 16 Aug1976, WAI 1100; New Zealand, Moanatuatua Bog, not flowering, K. Thompson 3/77, WAI 2100; New Zealand, Kaitaia, not flowering,W.F.B. Oliver, 26 Feb 1929, WELT19806; New Zealand, Ohaupo Swamp, ♂ flowers, T.F. Cheeseman, WELT19805; New Zealand, Ohaupo Swamp, not flowering, W. Petrie, WELT19804; New Zealand, Ohaupo Swamp, ♂ in flower, W. Petrie, WELT19803; New Zealand, Ohaupo Swamp, ♂ flowers, W. Petrie, WELT19802; New Zealand, Maitahi shrubland, not flowering, A.R Jamieson, AK231291; New Zealand, Rukuhia Swamp ♂ and ♀ plants in flower, L.M. Cranwell 18/34, AK109372; New Zealand, Lake Ohia, ♂ flowers, R. Cooper, R. Mason, N. Moar 1 Aug 1949, AK35820; New Zealand, Torehape Peat Dome ♂ flowers, A.E. Wright 10576, AK215859; New Zealand, Whangamarino Swamp, ♂ flowers, plants up to 1.2 m tall, E.K. Cameron 8839; AK234026; New Zealand, Kaihu Valley, ♂ flowers, A.R. Jamieson 30 Oct 1999, AK286616; New Zealand, Tomarata Lakes, not flowering, M.E. Young 20 March 2007, AK299780; New Zealand, Moanatuatua Peat Reserve, Rukuhia, ♂ flowers, F.J. Newhook July 1979, AK304253; New Zealand, Rukuhia Swamp, separate plants with ♂ and ♀ flowers, L.M. Cranwell 18/34, AK109373; New Zealand, Mangawhai Black Swamp, immature ♀flowers, M.E. Young 18 July 1999, AK239846; New Zealand, Mercer Swamp, P. Hynes 15 Feb 1964, AK101004; New Zealand, Tomarata Lakes, with few ♀ flowers, M.E. Young 20 March 2007, AK299780; New Zealand, Lake Ohia, ♂ flowers, A.E. Wright 10554, AK232056; New Zealand, Lake Ohia, ♀ flowers and fruits, A.E. Wright 10555, AK232055; New Zealand, Tokerau Beach, M.E. Young, L.J. Forester 17 Oct 2006, AK306920; New Zealand, Lake Ohia, ♂ flowers, J.E. Braggins 87/87A, AK304249.

The acronym for the University of Waikato herbarium was recently changed from WAI to WAIK. We cited the older WAI acronym which appeared on specimen labels that we studied.

#### Distribution.

New Zealand endemic ranging from North Cape southwards to approximately 38° S latitude.

#### Habitat.

*Empodisma robustum* is restricted to ombrotrophic raised peat bogs where it often coexists with *Sporadanthus ferrugineus*, fens and gumland heathland peats. Locally abundant, but populations becoming fragmented by intensive land use.

#### Conservation status.

Widespread drainage and conversion to pasture has dramatically reduced the extent of raised peat bogs in Northland and Waikato. This unique ecosystem is severely fragmented and provides habitat for a number of rare plant species such as *Sporadanthus ferrugineus* (de Lange et al. 1999) and *Dianella haematica* (Heenan and de Lange 2007). However, *Empodisma robustum* is still relatively common in shallower/younger peat systems, and probably does not yet qualify as a threatened species. We recommend that its conservation status be regularly reviewed.

### 2. Empodisma minus

(Hook.f.) L.A.S.Johnson & D.F.Cutler, Kew Bull. 28, 383 (1973)

http://species-id.net/wiki/Empodisma_minus

Calorophus minor Hook.f., *Fl. Nov. Zel. I*, 267 (1852–1853).
Calorophus elongatus var. *minor* (Hook.f.) Hook.f., *Fl. Tas. II*, 75 (1858–1859).
Hypolaena lateriflora var. *minor* (Hook.f.) Cheeseman, *Manual N.Z. Flora* ed. 1, 762 (1906)

#### Lectotype.

**Figure 10.**
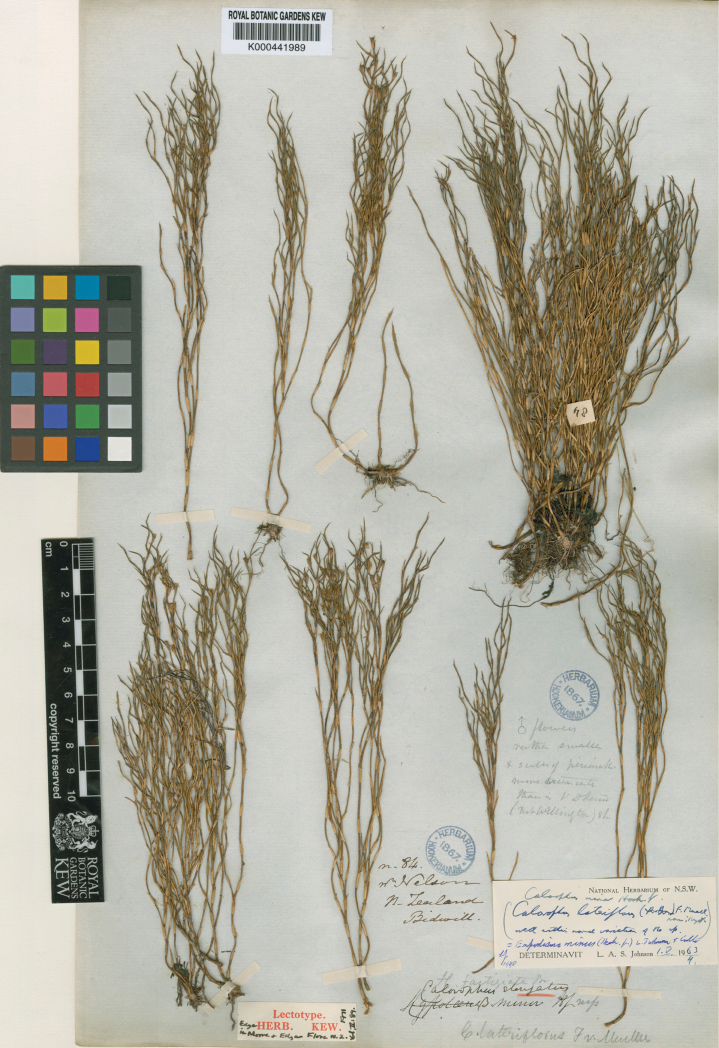
High resolution photograph of the lectotype of *Empodisma minus* (Hook.f.) L.A.S. Johnson & D.F.Cutler. Reproduced with the consent of the Royal Botanic Gardens, Kew, © The Board of Trustees of the Royal Botanic Gardens. Hooker (1853) described the new species *Calorophus minor* Hook.f. based upon Bidwell, Colenso and Lyall specimens. A specimen collected near Nelson by Bidwell, no. 84, K000441989, was chosen as the lectotype by Moore and Edgar (1970).

New Zealand, near Nelson, Bidwell no. 84, K000441989; (Fig. 10; designated by Moore and Edgar 1970, pg 89).

#### Etymology.

minus describes the small stature of *Empodisma minus*.

#### Description.

**Figure 11.**
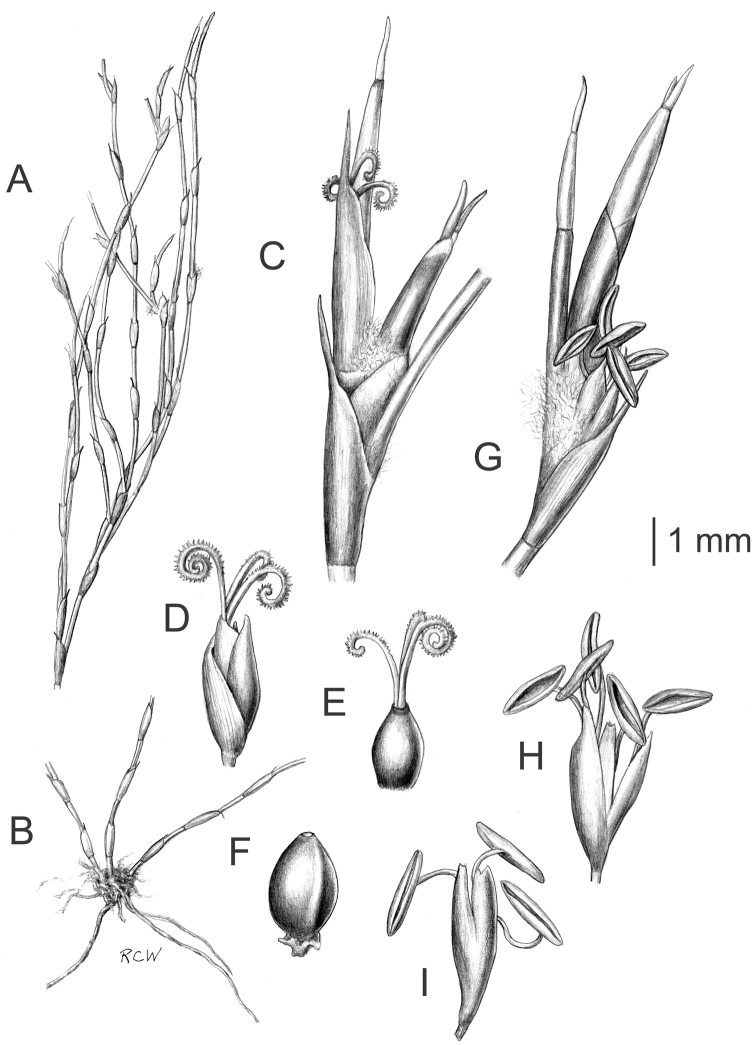
Morphological characteristics of *Empodisma minus*. **A** Vegetative shoot with attached leaves and flowers (2.5× actual size) **B** Rhizomes with emerging vegetative shoots (2.5× actual size) **C** Vegetative shoot with attached pistillate flower **D** Pistillate flower with attached bracts **E** Gynoecium **F** Mature nut **G** Vegetative shoot with attached staminate spikelet **H** Staminate flower with attached bracts **I** Staminate flower. Scale bar = 1 mm.

Culms dark green, 12–81 cm in height, 0.7–1.3 mm in diameter, branching profusely. Leaf sheaths closely appressed, 3.5–10.2 mm in length, borne at short intervals 15.0–48.0 mm; light green to light brown early in the season maturing dark brown; mouth ciliate with a prominent tuft of woolly white hairs in New Zealand specimens, spare or lacking in Australian specimens. Lamina strongly reflexed from leaf sheath, 1.5–4.2 mm long, persistent light green when young maturing dark brown. Spikelets brown, male spikelet 3.9–8 mm long, anthers 1.2–2.0 mm long; female spikelet 3.5–7.0 mm long; nutlets dark brown approximately 2.6 mm long. 2n = 24. Flowering Aug.– Apr. [Fig. 11; see also illustration in Meney and Pate (1999)].

#### Comments.

Morphologically similar to small forms of *Empodisma robustum* distinguished by its smaller stature (though plants from 0.4 to 1 m tall are noted from eastern Australia), more delicate culms and smaller spikelets. Most collections are sterile or male, and the few females generally lack mature fruits. Chromosome counts of 2n=24 were reported from plants collected from the NW slope of Mt. Ruapehu and three counts from NSW (Briggs 1966, Johnson and Cutler 1973).

####  Representative specimens.

Australia, Tasmania, near Margate, ♂ flowers, D.A. & A. Ratkowsky 1474, CHR303032, Australia, Queensland, Moreton Island, ♂ flowers, L. Durrington 1114 & S. Levine, CHR272564, Australia, Tasmania, Newdegate Pass, not in flower, T. Dobson 77107, CHR313744; New Zealand, S. Westland, ♂ flowers, G.C. Kelly, Oct 1966, CHR177206; New Zealand, Rahu Saddle, with few ♀ flowers, E.J. Godley, 1 July 1958, CHR108315; New Zealand, Bell Hill Plains, ♂ flowers, J. Clarke 1 Feb 1969, CHR189013; New Zealand, Ngamatea, ♂ flowers, N.J. Moar, 12 Jan 1949, CHR70144; New Zealand, Waikareiti, ♂ flowers, A.P. Druce, Feb 1968, CHR180674; New Zealand, Silica Springs Track, ♂ flowers G. Rennison, A61/36, , CHR535708; New Zealand, Makerikeri tarns, ♂ flowers, A.P. Druce, Nov 1973, CHR260376; New Zealand, Tussock Creek, ♂ flowers, L.B. Moore, 28 July 1968, CHR188099; New Zealand, Mokoreta, with few ♀ flowers, W.R. Sykes 41/94, CHR497058; New Zealand, Bayswater Bog, not flowering, B.R. Clarkson19 Feb .2009, CHR605146; New Zealand, Awarua Bay, not flowering, P.N. Johnson 653, CHR437892; New Zealand, West Cape, A.F. Mark, 5 Feb 1972, CHR218694; New Zealand, Coal Creek, ♂ flowers; I. Payton, 13 Sept 1976, CHR520808; New Zealand, Mt. Rockport, not flowering, I.A. McNew, 31 July 1942, CHR35234; New Zealand, Lake Sylvester, ♂ flowers, R. Melville 5915, CHR142781; New Zealand, Lake Sylvester, ♀ flowers, R. Mason & N. Moar 4658, CHR95709; New Zealand, Bealey spur, with immature ♀ flowers, P. Douglas 26 Nov 1979, CHR362302; New Zealand, Lake Tennyson, ♂ flowers, M.J.A. Simpson 6315, CHR22759; New Zealand, Patterson Inlet, ♂ flowers, L.J. Dumbleton & E. Edgar, CHR182509; New Zealand, ♂ flowers, Fosberg, Feb 1949; CHR30378; New Zealand, Kaitangata, plants with ♀ and ♂ flowers, R. Mason & N.T. Moar 953, CHR 75833; New Zealand, Kapuka, ♀ flowers, W.H. Harbond 20 Nov 1968, CHR183615.

#### Distribution.

Widely distributed in Tasmania and all mainland Australian States except Western Australia and the Northern Territory; in New Zealand extending north to approximately 38°S latitude.

#### Habitat.

Locally abundant in seasonally or permanently inundated wetlands, heathlands, fens and peat bogs from sea level to alpine

#### Conservation status.

Not threatened.

### 3. Empodisma gracillimum

(F.Muell.) L.A.S. Johnson & D.F.Cutler Kew Bull. 28: 383 (1973)

http://species-id.net/wiki/Empodisma_gracillimum

Calorophus gracillimus F. Muell., *Fragm. Phytogr. Australiae 8*, 88 (1872–74) as ‘*Calostrophus*’
Hypolaena gracillima (F.Muell.) Benth., *Fl. Austral. 7*, 239 (1878).

#### Syntypes.

**Figure 12.**
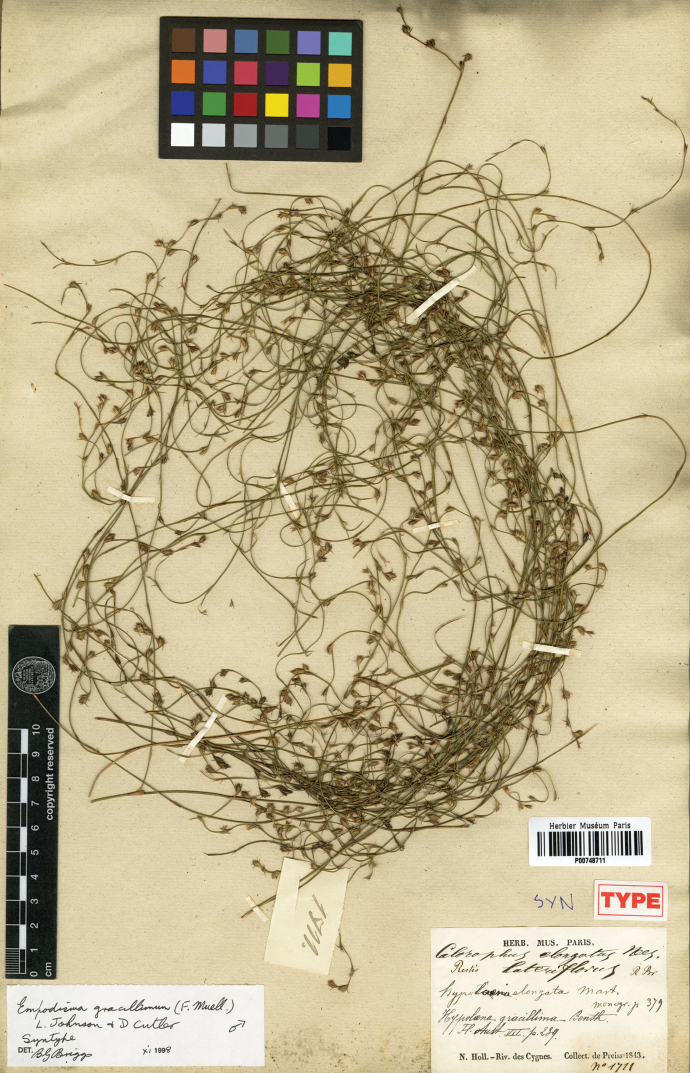
High resolution photograph of a syntype of *Empodisma gracillimum* (F.Muell.) L.A.S. Johnson & D.F.Cutler. Mueller (1872–74) originally described the plant as *Calorophus gracillimus* F.Muell. The specimen, Nouvelle-Hollande, Riv. des cygnes, Preiss JA 1711, 1843, P00748711 is held at HERBARIUM MUSEI PARISIENSIS. The syntype was designated by BG Briggs xi.1998.

(Fig. 12; *fide* BG Briggs xi.1998), Nouvelle-Hollande, Riv. des cygnes, Preiss JA 1711, 1843. P00748711; Nouvelle-Hollande, Riv. des cygnes, Preiss JA 1714, 1843. P00748712.

#### Etymology.

gracillimum describes the slender culms of *Empodisma gracillimum*.

#### Description.

(illustrated in Meney and Pate 1999). Culms delicate light green, 55–110 cm in height, 0.5–1.2 mm in diameter, branching profusely. Leaf sheaths, open, closely appressed, 3.5–9.0 mm in length, borne at intervals 25.0–80.0 mm; lamina strongly reflexed from leaf sheath, 2.4–5.0 mm, persistent, light green when young becoming straw coloured. Spikelets light brown, male spikelet 4–5.8 mm, anthers 0.6–1.0 mm long; female spikelet 1.5–2.4 long mm borne on pedicels up to 20 mm long; nutlets approximately 1.4–2.5mm long straw-coloured. 2n = 24. Flowering Aug.–Apr.

#### Comments.

Though it approaches *Empodisma robustum* in height, *Empodisma gracillimum* is readily distinguished by its more delicate light green culms and shorter leaf sheaths. The male and female spikelets of *Empodisma gracillimum* are smaller than either *Empodisma robustum* or *Empodisma minus*. The female spikelets are solitary and distinctly pedicillate; this character may be a synapomorphy for the species.

####  Representative specimens.

Western Australia,Denmark, ♂ and ♀ flowers, B.G. Briggs 8449 & L.A.S. Johnson, CHR525963; Western Australia,4.4 km east Watershed Road, ♂ flowers, A.R. Annels, R.W. Hearn 5112, PERTH04219031; Australia,4.4 km east Watershed Road, fruits, A.R. Annels, R.. Hearn 5111, PERTH04128567; Western Australia,Denmark, ♀ flowers, B.G. Briggs 8449 & L.A.S. Johnson, PERTH01586645; Western Australia,S. of junction with Brockman Highway, ♀ flowers D. Bright, C. Godden & T. Annels SC72.9 PERTH04723732; Western Australia, London Forest Block, 2km S of Mountain Road along Renzo Road, ♂ flowers, R.J. Cranfield & B.G. Ward WFM53, PERTH07102399; Western Australia,Torndirrup National Park, ♂ flowers, G.J. Keighery 8805, PERTH02182831; Western Australia,Darling, ♂ flowers, B.G. Briggs 9330, PERTH06173853; Western Australia,800 m E along O’Byrene Road from intersection of Commonage Road, ♂ flowers, N. Casson & T. Annels SC32.9, PERTH04741110; Western Australia,Walpole-Nornalup National Park, ♀ flowers, A.R. Annels ARA1580, PERTH05466172; Western Australia,Walpole, R.J. Cranfield 10897, PERTH04638530; Western Australia,600 m S of Brockman Highway on Beck Road, ♂ flowers, N. Casson, P. Ellery & C. McChesney SC74.8, PERTH04723775; Western Australia,400 m E of Blackwood and Great North Road, ♂ flowers, R. Davis 7680, PERTH05139317; Western Australia,WA, N. Casson & D. Bright SC106.2, PERTH04749677; Western Australia,S.E. Witchcliff, ♂ flowers, G.J. Keighery 16277, PERTH06330266; Western Australia,NE of Albany, E.M. Sandiford & D.A. Rathbone 1372, PERTH07926855.

#### Distribution.

Endemic to western Australia on the coastal plain south of Perth extending along the south coast from Augusta to Albany.

#### Habitat.

Grows on peat or sandy nutrient-poor soils. Locally abundant in seasonally or permanently inundated wetlands, swamps and stream margins.

## Supplementary Material

XML Treatment for Empodisma

XML Treatment for Empodisma robustum

XML Treatment for Empodisma minus

XML Treatment for Empodisma gracillimum

## Appendix 1

**Table:** Voucher specimens listing country of origin, literature citations, Allan Herbarium accession numbers for new sequences, their collection locality, DNA accession number, and GenBank accession numbers.

**Specie**	**Country**	**Literature citation**	**Locality**	**Herbarium accession**	**DNA accession**	*matK*	*rbcL*	*trnL*
*Alexgeorgea subterranean* Carlquist	Australia	Briggs et al. (2010)		NSW 437369		GQ409034	GQ408918	GQ408988
*Anarthria polyphylla* Nees	Australia	Briggs et al. (2000, 2010)		NSW 391527		DQ257498	AF148760	AF148720
*Apodasmia brownii* (Hook.f.) B.G.Briggs & L.A.S.Johnson	Australia	Briggs et al. (2010)		NSW 494422		—<br/>	GQ408919	—
*Apodasmia chilensis* (Gay) B.G.Briggs & L.A.S.Johnson	Chile	Wardle et al. (2001)	Los Lagos, Mehuín	CHR513924		JX154568	AF307923	JX154570
*Apodasmia similis* (Edgar) B.G.Briggs & L.A.S.Johnson	New Zealand	Wardle et al. (2001)	Westland, Wanganui River	CHR517317		JX154569	AF307924	JX154571
*Baloskion tetraphyllum* (Labill.) B.G.Briggs & L.A.S.Johnson	Australia	Briggs et al. (2000, 2010)		NSW365050		DQ257501	AF148761	AF148721
*Calorophus elongatus* Labill.	Australia	Briggs et al. (2000, 2010)		NSW 264835		GQ409036	DQ257502	AF148725
*Calorophus erostris* (C.B.Clarke) L.A.S.Johnson & B.G.Briggs	Australia	Briggs et al. (2000, 2010)		NSW 264698		GQ409036	GQ408930	GQ408999
*Chaetanthus aristatus* (R.Br.) B.G.Briggs & L.A.S.Johnson	Australia	Briggs et al. (2000, 2010)		NSW 261929		DQ257508	AF148782	AF148743
*Chordifex crispatus* (R.Br.) B.G.Briggs & L.A.S.Johnson	Australia	Briggs et al. (2000, 2010); Briggs and Johnson (2004)		NSW 401500		DQ257510	GQ408923	GQ408922
*Chordifex dimorphus* (R.Br.) B.G.Briggs	Australia	Briggs et al. (2000, 2010); Briggs and Johnson (2004)		NSW 270162		—	AF148763	AF148723
*Chordifex fastigiatus* (R.Br.) B.G.Briggs	Australia	Briggs et al. (2000, 2010); Briggs and Johnson (2004)		NSW 270160		—	AF148791	AF148752
*Chordifex hookeri* (D.I.Morris) B.G.Briggs,	Australia	Briggs et al. (2000); Briggs and Johnson (2004)		NSW 264839		—	AF148762	AF148722
*Coleocarya gracilis* S.T.Blake	Australia	Briggs et al. (2000, 2010)		NSW 401500		GQ409023	AF148769	AF148730
*Dapsilanthus ramosus* (R.Br.) B.G.Briggs & L.A.S.Johnson	Australia	Briggs et al. (2000, 2010)		NSW 338881		—	AF148780	AF148741
*Desmocladus castaneus* B.G.Briggs & L.A.S.Johnson	Australia	Briggs et al. (2000, 2010)		NSW 423447		DQ257511	AF148770	AF148731
*Dielsia stenostachya* (W.Fitzg.) B.G.Briggs & L.A.S.Johnson	Australia	Briggs et al. (2000, 2010)		NSW 391321		—	AF148771	AF148732
*Empodisma gracillimum* (F.Muell.) L.A.S. Johnson & D.F.Cutler	Australia	This paper	Western Australia, Denmark	CHR 525963	9.14	JX129074	JX129095	JX129133
*Empodisma gracillimum* (F.Muell.) L.A.S. Johnson & D.F.Cutler	Australia	This paper	Western Australia, Caldyannup land system	PERTH 07102399<br/>	12.1	JX129076	JX129095	JX129132
*Empodisma gracillimum* (F.Muell.) L.A.S. Johnson & D.F.Cutler	Australia	This paper	Western Australia, NE of Albany	PERTH 046385530<br/>	12.2	JX129075	JX129097	JX129131
*Empodisma minus* (Hook.f.) L.A.S.Johnson & D.F.Cutler	Australia	This paper	Tasmania, Central Plateau, second lagoon	CHR 585759	7.45	JX129080	JX129101	JX129116
*Empodisma minus* (Hook.f.) L.A.S.Johnson & D.F.Cutler	Australia	This paper	Victoria, Mt. Buffalo	CHR 607930	9.15	JX129090	JX129111	JX129117
*Empodisma minus* (Hook.f.) L.A.S.Johnson & D.F.Cutler	New Zealand	This paper	Waipapa EA, Pureora	CHR 605145	9.04	JX129087	JX129108	JX129119
*Empodisma minus* (Hook.f.) L.A.S.Johnson & D.F.Cutler	New Zealand	This paper	Lookout Range	CHR 605066	8.09	JX129085	JX129106	JX129120
*Empodisma minus* (Hook.f.) L.A.S.Johnson & D.F.Cutler	New Zealand	This paper	Herangi Range, top	CHR 596548	8.08	JX129084	JX129105	JX129121
*Empodisma minus* (Hook.f.) L.A.S.Johnson & D.F.Cutler	New Zealand	This paper	Taramoa	CHR 605065	8.01	JX129081	JX129102	JX129122
*Empodisma minus* (Hook.f.) L.A.S.Johnson & D.F.Cutler	New Zealand	This paper	Stewart Island, Mason Bay	CHR 605074	7.40	JX129077	JX129098<br/>	JX129123
*Empodisma minus* (Hook.f.) L.A.S.Johnson & D.F.Cutler	New Zealand	This paper	Pukerau Red Tussock Reserve	CHR 605847	9.05	JX129088	JX129109	JX129118
*Empodisma robustum* Wagstaff & Clarkson sp. nov.	New Zealand	This paper	Whangamarino	CHR 605067	11.01	JX129091	JX129112	JX129124
*Empodisma robustum* Wagstaff & Clarkson sp. nov.	New Zealand	This paper	Opuatia	CHR 605068	7.44	JX129079	JX129100	JX129129
*Empodisma robustum* Wagstaff & Clarkson sp. nov.	New Zealand	This paper	Moanatuatua Swamp	CHR 605165	9.06	JX129089	JX129110	JX129125
*Empodisma robustum* Wagstaff & Clarkson sp. nov.	New Zealand	This paper	Northland, Tangonge	CHR 605064	8.03	JX129082	JX129103	JX129128
*Empodisma robustum* Wagstaff & Clarkson sp. nov.	New Zealand	This paper	Lake Tomarata	CHR 605069	7.43	JX129078	JX129099	JX129130
*Empodisma robustum* Wagstaff & Clarkson sp. nov.	New Zealand	This paper	Kaimaumau	CHR 605063	8.06	JX129083	JX129104	JX129127
*Empodisma robustum* Wagstaff & Clarkson sp. nov.	New Zealand	This paper	Torehape Bog	CHR 605167	9.01	JX129086	JX129107	JX129126
*Eurychorda complanata* (R.Br.) B.G.Briggs & L.A.S.Johnson	Australia	Briggs et al. (2000, 2010)		NSW 264949		DQ257514	AF148790	AF148751
*Harperia lateriflora* W.Fitzg.	Australia	Briggs et al. (2000, 2010)		NSW 423455		GQ409020	AF148776	AF148737
*Hopkinsia adscendens* B.G.Briggs & L.A.S.Johnson	Australia	Briggs et al. (2000, 2010)		NSW 364372		DQ257519	AF148727	AF148738
*Hypolaena exsulca* R.Br.	Australia	Briggs et al. (2000, 2010)		NSW 364832		—	GQQ408927	—
*Hypolaena grandiuscula* F.Muell.	Australia	Briggs et al. (2000, 2010)		NSW 714757		—	—	GQ408962
*Hypolaena pubescens* (R.Br.) Nees	Australia	Briggs et al. (2000, 2010)		NSW 714454		GQ409046	—	GQ409963
*Hypolaena robusta* Meney & Pate	Australia	Briggs et al. (2000, 2010)		NSW 714451		—	—	GQ408964
*Kulinia eludens* B.G.Briggs & L.A.S.Johnson	Australia	Briggs et al. (2000, 2010)	<br/>	NSW 391535		—	AF148778	AF148739
*Lepidobolus chaetocephalus* Benth.	Australia	Briggs et al. (2000, 2010)		NSW 364813		—	AF148779	AF148740
*Leptocarpus tenax* (Labill.) R.Br.	Australia	Briggs et al. (2000, 2010)		NSW 264954		GQ409039	AF148781	AF148742
*Lepyrodia glauca* (Nees) F.Muell.	Australia	Briggs et al. (2000, 2010)		NSW 423726		—	AF148785	AF148746
*Loxocarya gigas* B.G.Briggs & L.A.S.Johnson	Australia	Briggs et al. (2000, 2010)		NSW 364738		—	AF148786	AF148747
*Lyginia barbata* R.Br.	Australia	Briggs et al. (2000, 2010)		NSW 391339		DQ257523	AF148787	AF148748
*Meeboldina coangustata* (Nees) B.G.Briggs & L.A.S.Johnson	Australia	Briggs et al. (2000, 2010)		NSW 261610		GQ409043	AF148284	AF148745
*Melanostachya ustulata* (F.Muell. ex Ewart & Sharman) B.G.Briggs & L.A.S.Johnson	Australia	Briggs et al. (2000, 2010)		NSW 232599		GQ409035	AF148788	AF148749
*Restio distichus* Rottb.	Africa	Moline and Linder (2005)		Linder, Hardy and Moline 7327		AY881540	AY881467	AY881613
*Sporadanthus ferrugineus* de Lange, Heenan & B.D.Clarkson	New Zealand	This paper	South Auckland; Moanatuatua Swamp	CHR 605163	9.10	JX129093	JX129114	JX129135
*Sporadanthus ferrugineus* de Lange, Heenan & B.D.Clarkson	New Zealand	This paper	South Auckland; Kopuati	CHR 604580	8.56	JX129092	JX129113	JX129134
*Sporadanthus gracilis* (R.Br.) B.G.Briggs & L.A.S.Johnson	Australia	Briggs et al. (2000, 2010)		NSW 270154		GQ409027	DQ257526	GQ409013
*Sporadanthus tasmanicus* (Hook.f.) B.G.Briggs & L.A.S.Johnson	Australia	Briggs et al. (2000, 2010)		NSW 264956		GQ409028	AF148793	AF148754
*Sporadanthus traversii* F.Muell.	New Zealand	This paper	Chatham Islands; Rakautahi	CHR 605164	9.08	JX129094	JX129115	JX129136
*Taraxis grossa* B.G.Briggs & L.A.S.Johnson	Australia	Briggs et al. (2000, 2010)		NSW 270154		GQ409027	DQ257526	GQ409013
*Tremulina tremula* B.G.Briggs & L.A.S.Johnson	Australia	Briggs et al. (2000, 2010)		NSW 264956		GQ409028	AF148793	AF148754
*Tyrbastes glaucescens* B.G.Briggs & L.A.S.Johnson	Australia	Briggs et al. (2000, 2010)		NSW 261641		GQ409037	AF148795	AF148756
*Winifredia sola* L.A.S.Johnson & B.G.Briggs	Australia	Briggs et al. (2000, 2010)		NSW 713239		GQ409021	AF148796	AF148758

## References

[B1] Agnew ADQ Wilson JB Sykes MT (1993) A vegetation switch as the cause of a forest/mire ecotone in New Zealand. Journal of Vegetation Science 4: 273-278 doi: 10.2307/3236115

[B2] Allan HH (1961) Flora of New Zealand. Vol. I. Government Printer, Wellington.

[B3] Barreda V Palazzesi L (2007) Patagonian vegetation turnovers during the Paleogene-early Neogene: origin of arid-adapted flora. The Botanical Review 73: 31-50 doi: 10.1663/0006-8101(2007)73[31:PVTDTP]2.0.CO;2

[B4] Bentham G (1878) Flora Australiensis, vol. 7. Reeve & Co., London.

[B5] Briggs BG (1966)Chromosome numbers of some Australian monocotyledons. Contributions of the N.S.W. National Herbarium IV: 24–34.

[B6] Briggs BG Marchant AD Gilmore S Porter CH (2000) A molecular phylogeny of Restionaceae and allies. In: Wilson KL Morrison DA (Eds). Monocots: Systematics and Evolution.CSIRO Press, Melbourne: 661-671

[B7] Briggs BG Johnson LAS (2004) New combinations in *Chordifex* (Restionaceae) from eastern Australia and new species from Western Australia. Telopea 10: 683-700

[B8] Briggs BG Marchant AD Perkins AJ (2010) Phylogeny and features in Restionaceae, Centrolepidaceae and Anarthriaceae (the restiid clade of Poales). In: Seberg O Petersen G Barford A Davis JI (Eds). Diversity, Phylogeny, and Evolution in the Monocotyledons.Aarhus University Press. Aarhus: 357-388

[B9] Briggs BG Linder PH (2009) A new subfamilial and tribal classification of Restionaceae (Poales). Telopea 12: 333-345

[B10] Brown R (1810) Prodromus Florae Novae Hollandiae et Insulae Van–Diemen. Taylor, London.

[B11] Burrows CJ Dobson AT (1972) Lakes Manapouri and Te Anau: mires of the Manapouri–Te Anau lowlands. Proceedings of the New Zealand Ecological Society 19: 75-94

[B12] Campbell DI Williamson JL (1997) Evaporation from a raised peat bog. Journal of Hydrology 193: 142-160 doi: 10.1016/S0022-1694(96)03149-6

[B13] Campbell EO (1964) The restiad peat bogs at Motumaoho and Moanatuatua. Transactions of the Royal Society of New Zealand: Botany 2: 219-227

[B14] Campbell EO (1975) Peat deposits of northern New Zealand as based on identification of plant fragments in the peat. Proceedings of the New Zealand Ecological Society 22: 57-60

[B15] Campbell EO (1983) Mires of Australasia. In: Gore AJP ed. Ecosystems of the World. Mires: swamp, bog, fen and moor. Elsevier, Amsterdam 153–180.

[B16] Campbell EO (1995) The significance of *Empodisma minus* (Restionaceae) in mires of eastern Australia, with particular reference to the coastal marshlands of SE Queensland. New Zealand Botanical Society Newsletter 42: 8-11

[B17] Cheeseman TF (1906) Manual of the New Zealand Flora. Government Printer, Wellington. doi: 10.5962/bhl.title.12003

[B18] Clarkson BR (1997) Vegetation recovery following fire in two Waikato peatlands at Whangamarino and Moanatuatua, New Zealand. New Zealand Journal of Botany 35: 167-179 doi: 10.1080/0028825X.1997.10414153

[B19] Clarkson BR (2002) Swamps, fens and bogs. In: Clarkson BD, Merrett M, Downs T, Botany of Waikato. Waikato Botanical Society, Hamilton, New Zealand.

[B20] Clarkson BR Schipper LA Clarkson BD (2004a) Vegetation and peat characteristics of restiad bogs on Chatham Island (Rekohu), New Zealand. New Zealand Journal of Botany 42: 293-312 doi: 10.1080/0028825X.2004.9512905

[B21] Clarkson BR Schipper LA Lehmann A (2004b) Vegetation and peat characteristics in the development of lowland restiad peat bogs, North Island, New Zealand. Wetlands 24: 133-151 doi: 10.1672/0277-5212(2004)024[0133:VAPCIT]2.0.CO;2

[B22] Clarkson BR Schipper LA Moyersoen B Silvester WB (2005) Foliar ^15^N natural abundance indicates phosphorus limitation in bog species. Oecologia 44: 550-557 doi: 10.1007/s00442-005-0033-4 15891854 10.1007/s00442-005-0033-4

[B23] Clarkson BR Schipper LA Silvester WB (2009) Nutritional niche separation in coexisting bog species demonstrated by ^15^N-enriched simulated rainfall. Austral Ecology 34: 377-385

[B24] Clarkson BR Smale MC Williams PA Wiser SK Buxton RP (2011) Drainage, soil fertility and fire frequency determine composition and structure of gumland heaths in northern New Zealand. New Zealand Journal of Ecology 35: 96–113

[B25] Cockayne L (1958) The Vegetation of New Zealand. J Cramer, London.

[B26] Cranwell LM (1939) Native vegetation. In Soils and Agriculture of part of Waipa County. DSIR Bulletin 76. Department of Scientific and Industrial Research, Wellington.

[B27] Crisp MD Cook LG (2007) A congruent molecular signature of vicariance across multiple plant lineages. Molecular Phylogenetic and Evolution 43: 1106-1117 doi: 10.1016/j.ympev.2007.02.030 10.1016/j.ympev.2007.02.030 17434758

[B28] Damman AWH (1978) Distribution and movement of elements in ombrotrophic peat bogs. Oikos 30: 480-495 doi: 10.2307/3543344

[B29] de Lange PR Heenan PB Clarkson BD Clarkson BR (1999) *Sporadanthus* in New Zealand. New Zealand Journal of Botany 37: 413-431 doi: 10.1080/0028825X.1999.9512645

[B30] DeQueiroz K (2007) Species concepts and species delimitation. Systematic Biology 56: 879-886 doi: 10.1080/10635150701701083 18027281 10.1080/10635150701701083

[B31] Drummond A Rambaut A (2007) BEAST: Bayesian evolutionary analysis by sampling trees. BMC Evolutionary Biology 7:214. doi: 10.1186/1471-2148-7-214 10.1186/1471-2148-7-214 PMC2247476 17996036

[B32] Farris JS Källersjö M Kluge AJ Bult C (1994) Testing significance of incongruence. Cladistics 10: 315-319 doi: 10.1111/j.1096-0031.1994.tb00181.x

[B33] Farris JS Källersjö M Kluge AJ Bult C (1995) Constructing a significance test for incongruence. Systematic Biology 44: 570-572

[B34] Felsenstein J (1985) Confidence limits on phylogenies: an approach using the bootstrap. Evolution 39: 783-791 doi: 10.2307/2408678 10.1111/j.1558-5646.1985.tb00420.x 28561359

[B35] Felsenstein J (1988) Phylogenies from molecular sequences: inferences and reliability. Annual Review of Genetics 22: 521-565 doi: 10.1146/annurev.ge.22.120188.002513 10.1146/annurev.ge.22.120188.002513 3071258

[B36] Givnish TJ Ames, M McNeal, JR McKain, MR Steele, PR dePamphilis CW Graham, SW Pires JC Stevenson DW Zomlefer WB Briggs BG Duvall MR Moore MJ Heaney JM Soltis DE Soltis PS Thiele K Leebens-Mack JH (2010) Assembling the tree of the monocotyledons: Plastome sequence phylogeny and evolution of Poales. Annals of the Missouri Botanical Garden 97: 584-616 doi: 10.3417/2010023

[B37] Hardy CR Linder PH (2005) Intraspecific variability and timing in ancestral ecology reconstruction: a test case from the Cape Flora. Systematic Biology 54: 299-316 doi: 10.1080/10635150590923317 16012098 10.1080/10635150590923317

[B38] Herendeen PS Crane PR (1995) The fossil history of the monocotyledons. In P. J. Rudall, P. J. Cribb, D. F. Cutler, and C. J. Humphries (ed.), Monocotyledons: systematics and evolution pp. 1–21. Royal Botanic Gardens, Kew, UK.

[B39] Hipp AL Hall JC Sytsma KJ (2004) Congruence versus phylogenetic accuracy: revisiting the incongruence length difference test. Systematic Biology 53: 81-89 doi: 10.1080/10635150490264752 14965902 10.1080/10635150490264752

[B40] Hodges TA Rapson GL (2011) Is *Empodisma* the ecosystem engineer of the FBT (fen-bog transition zone) in New Zealand? Journal of the Royal Society 40: 181–207.

[B41] Hooker JD (1852–1853) The Botany of the Antarctic Voyage of H.M. Discovery Ships Erebus and Terror in the Years 1839-1843.Vol. 2. Flora Novae-Zelandiae Part I. Flowering Plants. London, Lovell Reeve. 312 p.

[B42] Hooker JD (1858–1859) The Botany of the Antarctic Voyage of H.M. Discovery Ships Erebus and Terror in the Years 1839–1843.Vol. 3. Flora Tasmaniae Vol. II. Monocotyledones and Dicotyledones. London, Lovell Reeve. 422 p.

[B43] Hopper SD Gioia P (2004) The Southwest Australian Floristic Region: evolution and conservation of a global hot spot of biodiversity. Annual Review of Ecology, Evolution and Systematics 35: 623-650 doi: 10.1146/annurev.ecolsyst.35.112202.130201

[B44] Huson DH (1998) SplitsTree: analyzing and visualizing evolutionary data. Bioinformatics 14: 68-73 doi: 10.1093/bioinformatics/14.1.68 9520503 10.1093/bioinformatics/14.1.68

[B45] Huson DH Bryant D (2006) Application of phylogenetic networks in evolutionary studies. Molecular Biology and Evolution 23: 254-267 doi: 10.1093/molbev/msj030 16221896 10.1093/molbev/msj030

[B46] Johnson PN (2001) Vegetation recovery after fire on a southern hemisphere peatland. New Zealand Journal of Botany 39: 251-267 doi: 10.1080/0028825X.2001.9512736

[B47] Johnson PN Brooke PA (1989) Wetland Plants in New Zealand. DSIR, Wellington, New Zealand.

[B48] Johnson PN Gerbeaux P (2004) Wetland types in New Zealand. Department of Conservation, Wellington, New Zealand.

[B49] Johnson LAS Cutler DF (1973) *Empodisma*: a new genus of Australasian Restionaceae. Kew Bulletin 28: 381-385 doi: 10.2307/4108881

[B50] Kuder T Kruge MA Shearer JC Miller SL (1998) Environmental and botanical controls on peatification—a comparative study of two New Zealand restiad peat bogs using Py-GC/MS petrography and fungal analysis. International Journal of Coal Geology 37: 3-27 doi: 10.1016/S0166-5162(98)00022-6

[B51] Labillardière JJH de (1806)*Restio*, *Calorophus*. Novae Hollandiae Plantarum Specimen 2. Huzard, Paris, 77–79.

[B52] Lamont BB (1982) Mechanism for enhancing nutrient uptake in plants with particular reference to Mediterranean South Africa and Western Australia. Botanical Reviews 48: 597-689 doi: 10.1007/BF02860714

[B53] Linder PH Briggs BG Johnson LAS (1998) Restionaceae. In Kubitzki K (Eds) The Families and Genera of Flowering Plants. IV. Flowering Plants. Monocotyledons: Alismatanae and Commelinanae (except Gramineae). Berlin, Springer-Verlag. doi: 10.1146/annurev.ecolsys.36.102403.135635

[B54] Linder PH Eldenãs P Briggs BG (2003) Contrasting patterns of radiation in African and Australian Restionaceae. Evolution 57: 2688-2702 14761050 10.1111/j.0014-3820.2003.tb01513.x

[B55] Linder HP Rudall PJ (2005) Evolutionary History of Poales. Annual Review of Ecology, Evolution, and Systematics 36: 107-124

[B56] Mark AF Smith PMF (1975) A lowland vegetation sequence in South Westland: pakihi bog to mixed beech–podocarp forest. Part 1: the principal strata. Proceedings of the New Zealand Ecological Society 22: 76-92

[B57] McDougall KL (2007) Grazing and fire in two subalpine peatlands. Australian Journal of Botany 55: 42-47 doi: 10.1071/BT06096

[B58] McGlone MS (1985) Plant biogeography and the late Cenozoic history of New Zealand. New Zealand Journal of Botany 23: 723-749 doi: 10.1080/0028825X.1985.10434240

[B59] McGlone MS (2009) Postglacial history of New Zealand wetlands and implications for their conservation. New Zealand Journal of Ecology 33: 1-23

[B60] McPhail MK (1997) Comment of M Pole (1994): the New Zealand flora—entirely long-distance dispersal. Journal of Biogeography 22: 625-635 doi: 10.1071/BT96028

[B61] Meney KA Pate JS (Eds) (1999) Australian Rushes—Biology, Identification and Conservation of Restionaceae and Allied Families. University of Western Australia Press, Perth.

[B62] Meney KA Dixon KW Pate JS (1997) Reproductive potential of obligate seeder and resprouter herbaceous perennial monocots (Restionaceae, Anarthriaceae, Ecdeiocoleaceae) from South-western Western Australia. Australian Journal of Botany 45: 771-782

[B63] Merton PJ (1986) Investigation of two pakihi mires in South Westland. BSc dissertation, Christchurch, New Zealand: University of Canterbury. 69 pp.

[B64] Moline PM Linder HP (2005) Molecular phylogeny and generic delimitation in the *Elegia* group (Restionaceae, South Africa) based upon complete taxonomic sampling and four chloroplast DNA markers. Systematic Botany 30: 759-772 doi: 10.1600/036364405775097842

[B65] Mildenhall DC (1980) New Zealand late Cretaceous and Cenozoic plant biogeography: a contribution. Palaeogeography, Palaeoclimatology, Palaeoecology 31: 197-233 doi: 10.1016/0031-0182(80)90019-X

[B66] Moore LB Edgar E (1970) Flora of New Zealand II. Government Printer, Wellington, New Zealand.

[B67] Morrison DA (2006) Multiple sequence alignment for phylogenetic purposes. Australian Systematic Botany 19: 479-539 doi: 10.1071/SB06020

[B68] Mueller F (1872–1874) Fragmenta Phytographiæ Australiæ. Vol. 8. Government Printer, Melbourne.

[B69] Newnham RM de Lange PJ Lowe DJ (1995) Holocene vegetation, climate, and history of a raised bog complex, northern New Zealand based on palynology, plant macrofossils, and tephrochronology. The Holocene 5: 267-282 doi: 10.1177/095968369500500302

[B70] Pate JS Meney KA Dixon KW (1991) Contrasting growth and morphological characteristics of fire-sensitive (obligate seeder) and fire-resistant (resprouter) species of Restionaceae (S. Hemisphere restiads) from south-western Australia. : 505-525 doi: 10.1071/BT9910505

[B71] Pate JS Meney KA Dixon KW Bell TL Hickman EJ (1999) Response of Restionaceae to fire. In: Meney KA, Pate JS (Eds) Australian Rushes — Biology, Identification and Conservation of Restionaceae and Allied Families. University of Western Australia Press, Perth.

[B72] Posada D (2008) jModelTest: phylogenetic model averaging. Molecular Biology and Evolution 25: 1253-1256 doi: 10.1093/molbev/msn083 18397919 10.1093/molbev/msn083

[B73] Posada D Buckley TR (2004) Model selection and model averaging in phylogenetics: advantages of Akaike information criterion and Bayesian approaches over likelihood ratio tests. Systematic Biology 53: 793-808 doi: 10.1080/10635150490522304 15545256 10.1080/10635150490522304

[B74] Rambaut A (2009) FigTree v1.3.1. Computer program available from http://tree.bio.ed.ac.uk/software/figtree/

[B75] Rambaut A Drummond AJ (2007) Tracer v1.5. Computer program available from: http://tree.bio.ed.ac.uk/software/tracer/

[B76] Ramirez MJ (2006) Further problems with the incongruence length difference test: “hypercongruence” effect and multiple comparisons. Cladistics 22: 289-295

[B77] Rigg HH (1962) The pakihi bogs of Westport, New Zealand. Transactions of the Royal Society of New Zealand: Botany 1: 91-108

[B78] Scholz A (1985) The palynology of the upper lacustrine sediments of the Arnot Pipe, Banke, Namaqualand. Annals of the South African Museum 95: 1-109

[B79] Slatkin M Maddison WP (1981) A cladistic measure of gene flow inferred from the phylogenies of alleles. Genetics 103: 603-613 10.1093/genetics/123.3.603 PMC1203833 2599370

[B80] Sork VL Nason J Campbell DR Fernandez JF (1999) Landscape approaches to historical and contemporary gene flow in plants. Trends in Ecology and Evolution 14: 219-224 doi: 10.1016/S0169-5347(98)01585-7 10354623 10.1016/s0169-5347(98)01585-7

[B81] Swofford DL (2002) PAUP*: Phylogenetic Analysis Using Parsimony (and Other Methods). Sinauer Associates, Sunderland, MA.

[B82] Timmins SM (1992) Wetland vegetation recovery after fire: Eweburn Bog, Te Anau, New Zealand. New Zealand Journal of Botany 30: 383-399 doi: 10.1080/0028825X.1992.10412918

[B83] Thiers B [continuously updated] Index Herbariorum: A global directory of public herbaria and associated staff. New York Botanical Garden’s Virtual Herbarium. http://sweetgum.nybg.org/ih/

[B84] Thompson JD Gibson TJ Plewniak F Jeanmougin F Higgins DG (1997) The Clustal X windows interface: flexible strategies for multiple sequence alignment aided by quality analysis tools. Nucleic Acids Research 25: 4876-4882 doi: 10.1093/nar/25.24.4876 9396791 10.1093/nar/25.24.4876 PMC147148

[B85] Thompson MA Campbell DI Spronken-Smith RA (1999) Evaporation from natural and modified raised peat bogs in New Zealand. Agricultural and Forest Meteorology 95: 85-98 doi: 10.1016/S0168-1923(99)00027-1

[B86] Truswell EM Macphail MK (2009) Polar forests on the edge of extinction: what does the fossil spore and pollen evidence from East Antarctica say? Australian Systematic Botany 22: 57–106. doi: 10.1071/SB08046

[B87] Wahren C-HA Walsh NG (2000) Impact of fire in treeless subalpine vegetation at Mt Buffalo National Park, 1982–1999. Unpublished report to the Australian Alps Liaison Committee by La Trobe University, Melbourne.

[B88] Walsh NG McDougall KL (2004) Progress in the recovery of the flora of tress subalpine vegetation in Kosciuszko National Park after the 2003 fires. Cunninghamia 8: 439-452

[B89] Wardle P Ezcurra C Ramírez C Wagstaff SJ (2001) Comparison of the flora of the southern Andes and New Zealand. New Zealand Journal of Botany 39: 69-108 doi: 10.1080/0028825X.2001.9512717

[B90] Weins JJ Morril MC (2011) Missing data in phylogenetic analysis: reconciling results from simulations and empirical data. Systematic Biology 60: 719-731 doi: 10.1093/sysbio/syr025 21447483 10.1093/sysbio/syr025

[B91] Whinam J Hope GS (2005) The peatlands of the Australasian Region. In: Steiner GM (Ed). Mires. From Siberia to Tierra del Fuego.Stapfia 85: 397-433

[B92] Whinam J Kirkpatrick JB (1995) Successional sequences in two Tasmanian valley sphagnum peatlands. Journal of Vegetation Science 6: 675-682 doi: 10.2307/3236437

[B93] Yule GU (1924) A mathematical theory of evolution based on the conclusions of Dr. J.C. Willis. Philosophical Transaction of the Royal Society. 213: 21-87 doi: 10.1098/rstb.1925.0002

